# Heterologous pathway assembly reveals molecular steps of fungal terreic acid biosynthesis

**DOI:** 10.1038/s41598-018-20514-x

**Published:** 2018-02-01

**Authors:** Chuixing Kong, Hezhou Huang, Ying Xue, Yiqi Liu, Qiangqiang Peng, Qi Liu, Qin Xu, Qiaoyun Zhu, Ying Yin, Xiangshan Zhou, Yuanxing Zhang, Menghao Cai

**Affiliations:** 1 State Key Laboratory of Bioreactor Engineering, East China University of Science and Technology, 130 Meilong Road, Shanghai, 200237 China; 2 Shanghai Collaborative Innovation Center for Biomanufacturing, 130 Meilong Road, Shanghai, 200237 China

## Abstract

Terreic acid is a potential anticancer drug as it inhibits Bruton’s tyrosine kinase; however, its biosynthetic molecular steps remain unclear. In this work, the individual reactions of terreic acid biosynthesis were determined by stepwise pathway assembly in a heterologous host, *Pichia pastoris*, on the basis of previous knockout studies in a native host, *Aspergillus terreus*. Polyketide synthase AtX was found to catalyze the formation of partially reduced polyketide 6-methylsalicylic acid, followed by 3-methylcatechol synthesis by salicylate 1-monooxygenase AtA-mediated decarboxylative hydroxylation of 6-methylsalicylic acid. Our results show that cytochrome P450 monooxygenase AtE hydroxylates 3-methylcatechol, thus producing the next product, 3-methyl-1,2,4-benzenetriol. A smaller putative cytochrome P450 monooxygenase, AtG, assists with this step. Then, AtD causes epoxidation and hydroxyl oxidation of 3-methyl-1,2,4-benzenetriol and produces a compound terremutin, via which the previously unknown function of AtD was identified as cyclooxygenation. The final step involves an oxidation reaction of a hydroxyl group by a glucose-methanol-choline oxidoreductase, AtC, which leads to the final product: terreic acid. Functions of AtD and AtG were determined for the first time. All the genes were reanalyzed and all intermediates and final products were isolated and identified. Our model fully defines the molecular steps and corrects previous results from the literature.

## Introduction

**Figure 1:**
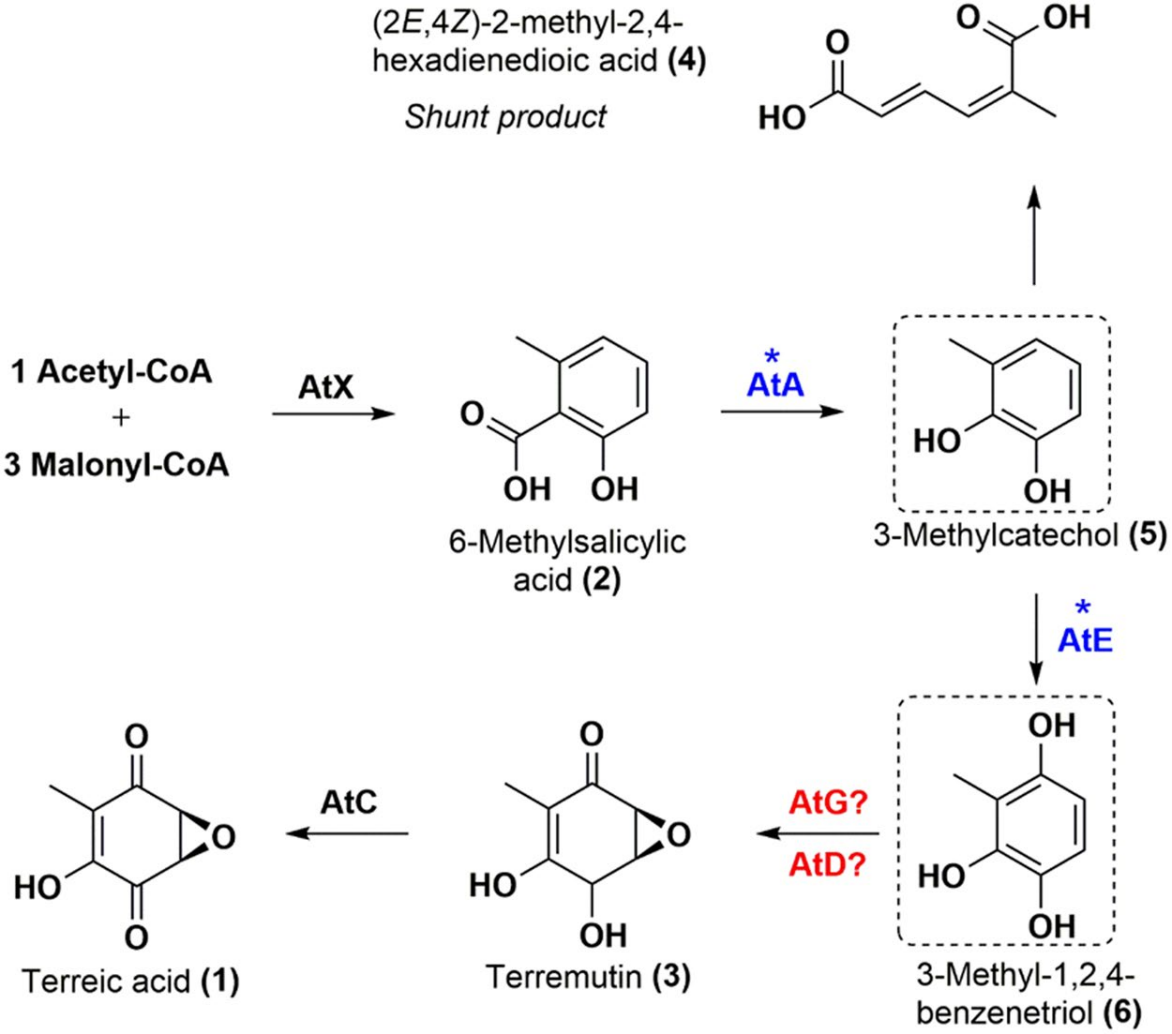
The proposed biosynthetic pathway of terreic acid (TA) in A. terreus. The scheme was referred to ref.^7^. Hypothetical compounds are boxed. Proteins and intermediates for TA biosynthesis are shown. Proteins with an unknown function are marked with a question mark, and proteins whose functions needed to be verified are marked with an asterisk.

Fungal secondary metabolites are well known for their wide-ranging biological activities. Terreic acid (TA, compound 1, Fig. 1) is a polyketide that was originally isolated from *Aspergillus terreus* and has an inhibitory effect against bacteria^1^. The compound also selectively inhibits the catalytic activity of Bruton’s tyrosine kinase (Btk), and this kinase significantly affects mast cell activation and B-cell development^2^. Recently, a selective inhibitor of Btk, ibrutinib, was approved by the US FDA for the treatment of mantle cell lymphoma and chronic lymphocytic leukemia^3^. Thus, TA or its derivative Btk inhibitors have a good potential as anticancer pharmaceuticals and arouse interest at present; characterization of TA’s biosynthetic mechanism will facilitate industrial biosynthesis and experiments with TA or screening of its bioactive derivatives.

Although a radiolabeled-precursor approach already demonstrated in the 1960s that TA derives from 6-methylsalicylic acid (6-MSA) via decarboxylation and a series of oxidation steps^4,5^, the whole sequential biosynthetic pathway has not been deciphered until recently. In 2014, Boruta and Bizukojc reported an *at* gene cluster for TA biosynthesis by bioinformatic analysis of *A*. *terreus* genome^6^. Guo and coworkers then proposed a biosynthetic pathway for TA by means of a gene knockout approach to this *at* cluster^7^. The 6-MSA synthase (6-MSAS) encoded by *atX*, which was identified by Fujii and coworkers for the first time^8^, first utilizes one acetyl-CoA as a starter unit and three malonyl-CoA molecules as extension units and catalyzes a series of programmed reactions including Claisen condensation, dehydration, reduction, and cyclization to generate 6-MSA (compound **2**, Fig. 1). The *atA*-encoded 6-MSA decarboxylase then catalyzes decarboxylation and hydroxylation reactions to form a predicted compound: 3-methylcatechol (compound **5**, Fig. 1), followed by a hydroxylation reaction catalyzed by the *atE*-encoded cytochrome P450 monooxygenase to produce a predicted compound, 3-methyl-1,2,4-benzenetriol (compound **6**, Fig. 1). This reaction could be catalyzed by a catechol 1,2-dioxygenase encoded by a gene outside the *at* cluster resulting in formation of a nonaromatic compound: (2*E*,4*Z*)-2-methyl-2,4-hexadienedioic acid (compound **4**, Fig. 1). Another cytochrome P450 monooxygenase, the one encoded by *atG*, was predicted to drive the next step (epoxidation) generating terremutin (compound **3**, Fig. 1). Then, a glucose-methanol-choline (GMC) oxidoreductase encoded by *atC* is thought to catalyze a reaction of oxidation of terremutin, thereby yielding the final product: TA^7^. Nevertheless, because of the complicated metabolic background in native *A*. *terreus*, compounds **5** and **6** could not be isolated from the strain and identified^7^. Although deficiency in *atE* leads to accumulation of a shunt product^7^, the relation between AtE and its substrate 3-methylcatechol still needs to be verified. Moreover, deficiency in *atD* and *atG* blocks TA synthesis but no intermediates or shunt products have been identified, leaving a gap in functional characterization of both enzymes^7^. Of note, AtD shows homology to PatJ from the patulin cluster^9^, but the functions of both putative enzymes are still unknown^7^; however, no characterized homologue can be identified for AtG even though it contains a conserved cytochrome P450 monooxygenase domain^7^.

To exactly determine the functions of AtA, AtE, AtG, AtD, and AtC, a stepwise pathway assembly in a heterologous host may work. In a previous study, we successfully constructed a *Pichia pastoris* (*Komagataella phaffii*) strain carrying *A*. *terreus atX* encoding 6-MSA synthase (6-MSAS)^10^. When *A*. *nidulans npgA* is introduced next, which encodes a phosphopantetheinyl transferase (PPTase) for activation of acyl carrier protein (ACP) domain in polyketide synthase (PKS), the target polyketide product 6-MSA is efficiently synthesized (2.2 g/L)^10^. The high activity of 6-MSAS in *P*. *pastoris* indicates that this host may be a suitable chassis organism for proteins from *A*. *terreus*, and it was therefore chosen for heterologous expression of the TA pathway in the present study.

Guo *et al*. reported that eight genes in the *at* cluster participate in TA biosynthesis, and *atB* located there was proposed to be a putative gene for a transporter, whereas *atF* was suggested to encode a putative zinc family transcription factor^7^. Because heterologous pathway assembly usually involves promoters and transcription factors from a chassis microorganism, *atF* is not necessary for the TA pathway expression in *P*. *pastoris*. The transporter protein is also dispensable for the TA synthesis process regardless of the producing ability. Besides, functions of *atX* were fully characterized by both a knockout^7^ and *P*. *pastoris* expression^10^ elsewhere. Thus, we mainly focused on *atA*, *atC*, *atD*, *atE*, and *atG* in this study. The functional genes of the *at* cluster were expressed here separately and combinatorially to clarify their roles in TA biosynthetic steps. The heterologous biosynthesis of TA and of the intermediates was realized via combinatorial expression of various functional genes, and the functions of the biosynthetic genes were finally confirmed and redefined, thereby correcting previous results from the literature and describing all the reactions of the TA biosynthesis pathway.

## Results

### Cloning and intron identification of TA biosynthetic genes

The mRNAs of *atA*, *atE*, *atG*, *atD*, and *atC* were obtained, reversely transcribed to cDNA and sequenced. The protein-coding sequence and introns of each gene were then identified (Supplementary Fig. S1), thus correcting the previous results of genome shotgun sequencing (GenBank accession No. CH476602.1). The newly identified sequences were deposited in the database, and details for each gene are summarized in Table 1. The full-length protein-coding sequence of *atA* is 1405 bp with an intron of 64 bp (Table 1), which codes for a protein of 466 amino acid residues (aa) and is different from that of the previously predicted sequence (gene locus: ATEG_06272). The full-length coding sequence of *atE* is 1850 bp [with four introns: 64, 61, 64, and 53 bp (Table 1)] coding for a 535 aa protein. Moreover, the identified *atE* was found to have 329 more base pairs in the 5′-flanking region as compared to that of the previously predicted sequence (gene locus: ATEG_06277). Repeated cloning and sequencing experiments showed that the *atD* (gene locus: ATEG_06276) previously predicted by whole-genome shotgun sequencing misses a cytosine nucleoside at position 1163. Thus, the coding sequence of *atD* is 1160 bp [with two introns 65 and 54 bp (Table 1)] encoding a 320 aa protein. The full-length coding sequence of *atG* is 583 bp [with two introns, 50 and 59 bp (Table 1)] coding for a 157 aa protein. The identified *atE* has 94 more base pairs in the 5′-flanking region as compared to that of previously predicted *atE* (gene locus: ATEG_06280). The full-length coding region of *atC* is 2277 bp [with eight introns: 52, 57, 58, 56, 50, 73, 60, and 50 bp (Table 1)] coding for a 606 aa protein, which is different from that of previously predicted *atC* (gene locus: ATEG_06274). The genes after intron removal were cloned from their cDNA and then applied to construction of expression plasmids.

**Table 1:** Gene characteristics and functions in the *at* gene cluster.

*at* gene cluster (ATEG_062XX.1)^7^					

Gene	*CDS length* /Intron(s) position	GenBank accession No.	Cofactors (putative)	Protein blast homologues (% identity, GenBank or UniProtKB accession No.)^a^	Function assigned
*atA*	*1405 bp/*847–910	KY950680	FAD/ NADH	NahG (34%, P23262.4)^13^; OpS4 (25%, J4VWM7)^14^; SalA (32%, AAG33865.1)^15^	salicylate 1- monooxygenase
*atE*	1850 *bp/* 140–203; 268–328; 706–769; 1191–1243	KY950681	NADH/ NADPH	PatI (63%, A1CFL6.2)^20^; PatH (54%, A1CFL5.1)^20^	cytochrome P450 monooxygenase
*atD*	*1160 bp/* 81–145; 597–650	KY950682	NADH/ NADPH	Cupin (70%, CDM36381.1)^21^; PatJ (62%, A1CFL7.1)^9^	epoxidase
*atG*	*583 bp*/ 142–191; 353–411	KY950683	NADH/ NADPH	unknown	cytochrome P450 monooxygenase
*atC*	*2277 bp/* 364–415; 469–525; 556–613; 1125–1180; 1292–1341; 1490–1562; 1989–2048; 2184–2233	KY950684	FAD	VBS (41%, AAC49318.1)^26,27^; VBS (41%, XP_002379930.1)^28^	GMC oxidoreductase

^a^Cupin and PatJ are not characterized.

### Identification of functions of AtX, AtA, and AtE

**Figure 2:**
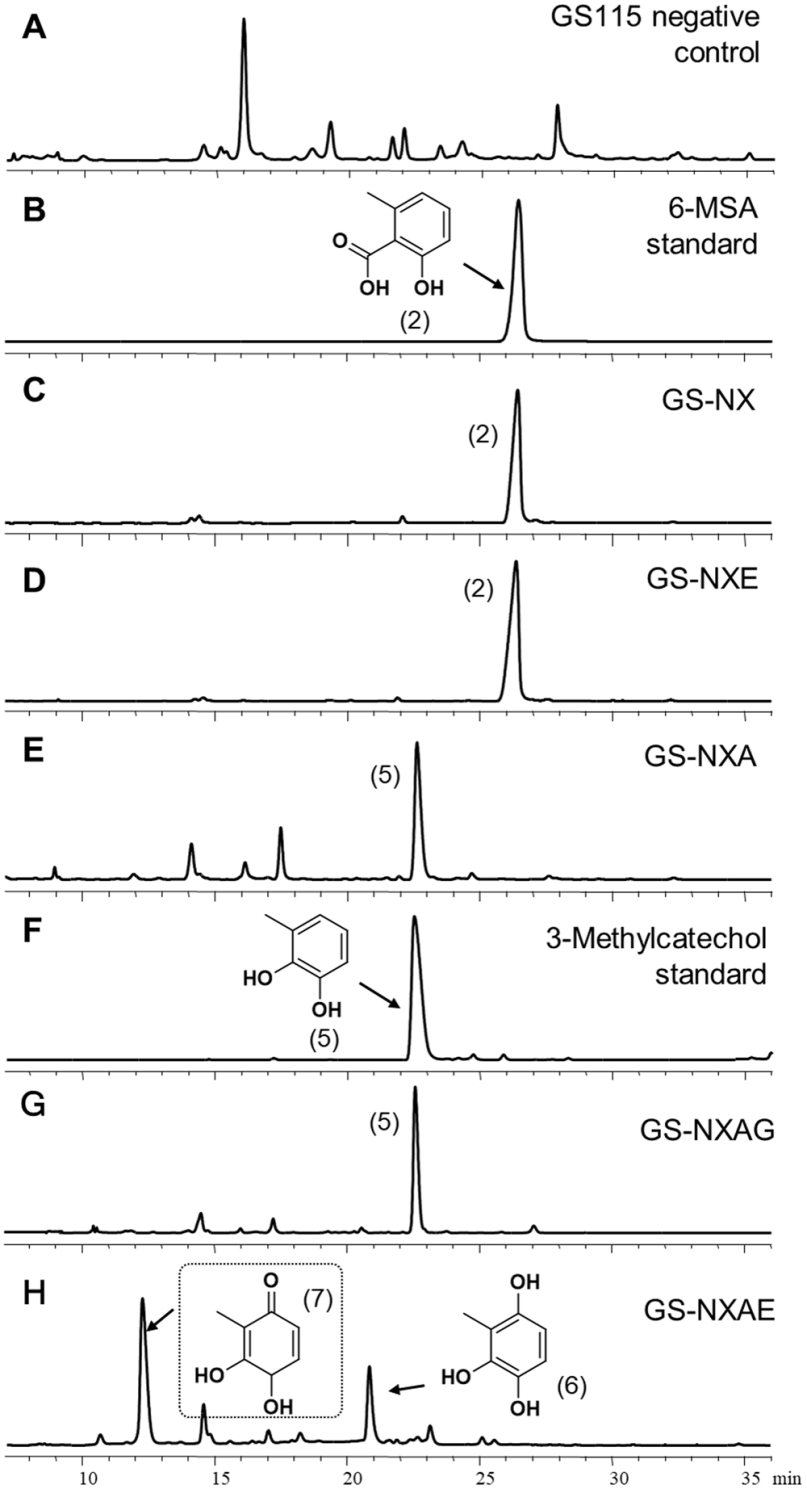
The HPLC chromatograms of organic extracts from culture broth. **(A)** Wild-type GS115 (negative control); **(B)** the 6-MSA standard; **(C)** strain GS-NX; **(D)** strain GS-NXE; **(E)** strain GS-NXA; **(F)** 3-methylcatechol standard; **(G)** strain GS-NXAG; **(H)** strain GS-NXAE. For HPLC, samples extracted from culture broth after methanol induction for 48 h were analyzed for UV absorbance at 254 nm. Chemical structure of boxed compound was inferred.

In our preliminary study, the expression of phosphopantetheinyl transferases encoded by *Aspergillus nidulans npgA* in *P*. *pastoris* turned inactive *apo*-ACP into active *holo*-ACP, leading to activation of polyketide synthase AtX and production of 6-MSA^10^. In this case, *npgA* and *atX* were reconstructed in a single plasmid (pPICβ-npgA-atX) to leave more selective markers for screening of various expression strains. Accordingly, strain GS-NX expressing *atX* and *npgA* under the control of promoter *P*_*AOX*1_ was constructed. Relevant products were extracted and analyzed by high-performance liquid chromatography (HPLC) after methanol induction for 48 h. Strain GS-NX produced 6-MSA (**2**) (193.6 mg/L) as compared to the wild-type GS115 strain (negative control; Fig. 2A–C). We next introduced *atA* or *atE* into strain GS-NX using the same promoter system. Analysis of products showed that the expression of *atA* resulted in a strain (GS-NXA) capable of producing 3-methylcatechol (**5**) (61.0 mg/L), whereas nothing changed after the expression of *atE* in GS-NX (Fig. 2D–F). The product of 3-methylcatechol was identified by liquid chromatography with mass spectrometry (LC-MS) and ^1^H nuclear magnetic resonance (NMR) analysis (Supplementary Fig. 2) in a comparison with other experimental results^11^. These findings proved that AtA but not AtE catalyzes the decarboxylative hydroxylation after AtX in TA biosynthesis, in agreement with gene knockout results in a native strain^7^. We then coexpressed *atE* and *atA* in GS-NX under the control of promoter *P*_*AOX*1_ and generated strain GS-NXAE. A comparison strain, GS-NXAG, was also created by expressing *atG* in GS-NXA because both AtE and AtG contain a conserved putative P450 monooxygenase domain (Table 1). Of note, strain GS-NXAE was found to produce two specific products (compound 6, 77.8 mg/L, and compound **7**, unstable [titer not determined]; Fig. 2H and E) whereas GS-NXAG does not produce any new compound (still generates compound **5**, 63.3 mg/L) as compared to GS-NXA (Fig. 2G and E). Next, compound **6** was isolated and analyzed for its chemical structure. Because no published NMR data are available for compound **6**, one-dimensional (1D) and 2D NMR spectroscopy (^1^H NMR, ^13^C NMR, heteronuclear multiple bond correlation [HMBC], and heteronuclear single quantum correlation [HSQC]) enabled us to identify its structure as 3-methyl-1,2,4-benzenetriol (m/z 140, Supplementary Fig. S3), which has not been previously isolated and identified^7^. Moreover, compound **6** was partially converted to a new compound, **7** (m/z 140 by LC-MS, ~12 min) with broth pH decreasing during strain GS-NXAE culture (Supplementary Fig. S4). Moreover, preparative–HPLC–purified compound **7** immediately transformed into **6** according to analytical HPLC assay (Supplementary Fig. S4). These results revealed that **6** and **7** have identical molecular weights and easily convert into each other; these data helped us to predict compound **7** as a tautomer of **6**. It is probably 3,4-dihydroxy-2-methylcyclohexa-2,5-diene-1-one, which is unstable and could not be purified for chemical structure analysis.

### Identification of functions of AtD, AtG, and AtC

**Figure 3:**
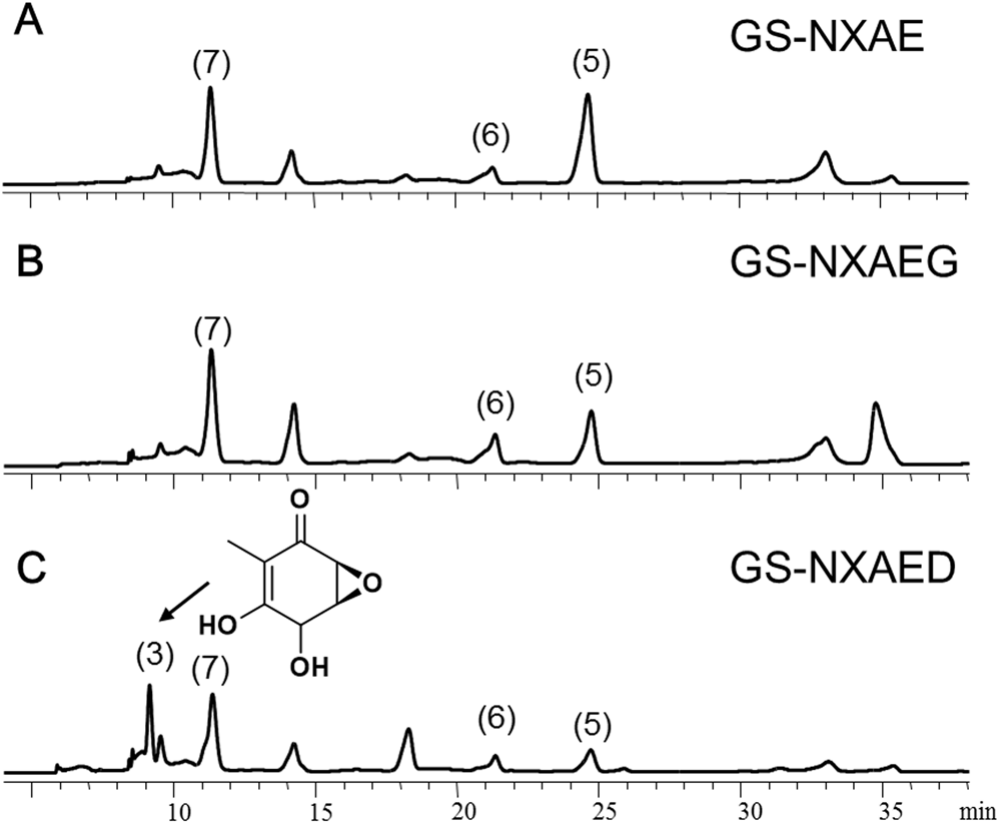
The HPLC chromatograms of organic extracts from culture broth. **(A)** Strain GS-NXAE; **(B)** strain GS-NXAEG; **(C)** strain GS-NXAED. For HPLC, samples were extracted from culture broth after methanol induction for 48 h and were analyzed for UV absorbance at 254 nm.

**Figure 4:**
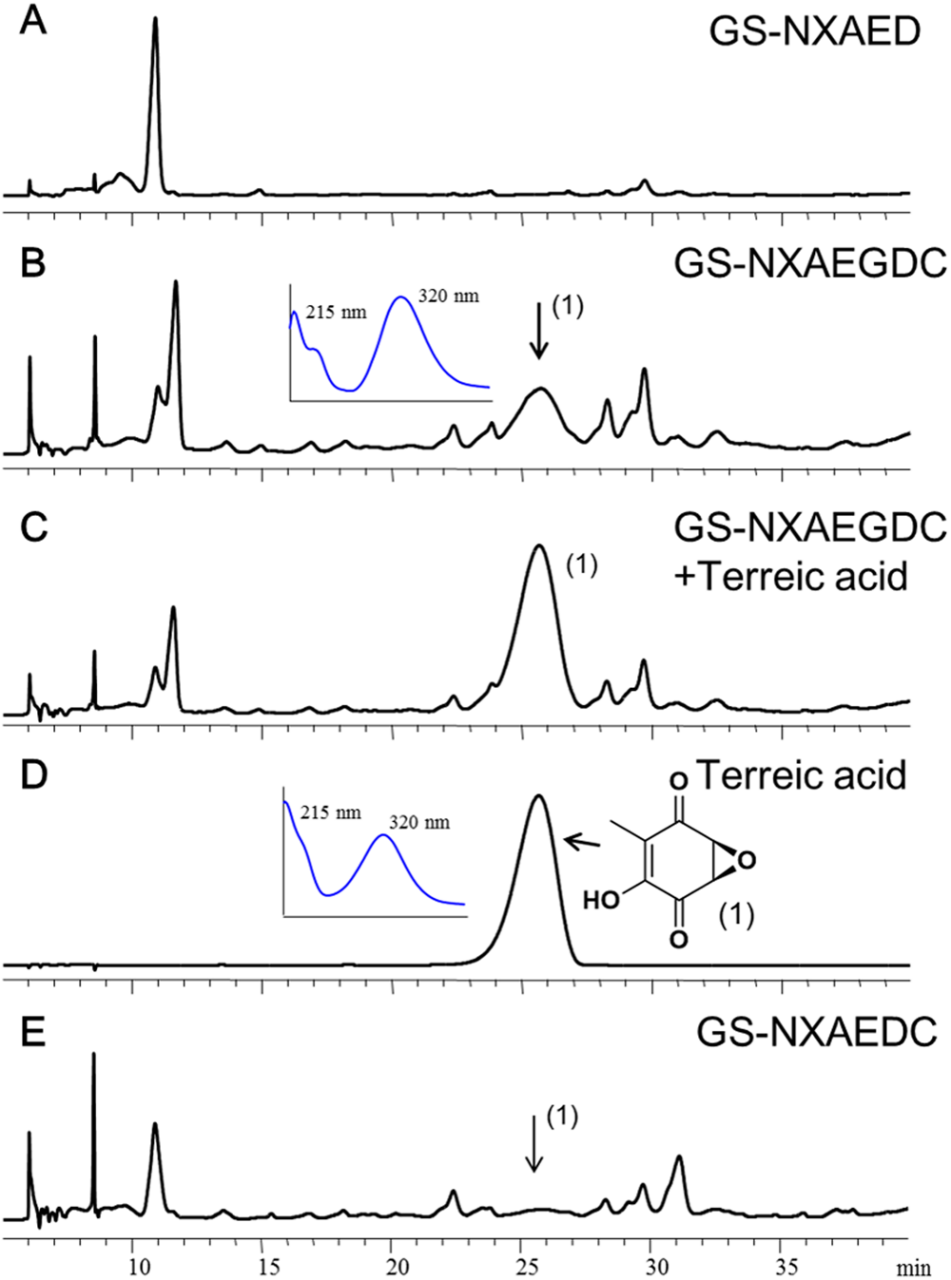
The HPLC chromatograms of organic extracts from culture broth. **(A)** Strain GS-NXAED; **(B)** strain GS-NXAEGDC; **(C)** strain GS-NXAEGDC supplemented with a terreic acid (TA) standard; **(D)** TA standard; **(E)** strain GS-NXAEDC. A UV spectrum of the specific peak and TA standard are shown. For HPLC, samples extracted from culture broth after methanol induction for 48 h were analyzed for UV absorbance at 330 nm.

Guo *et al*. knocked out *atG* and *atD* in native *A*. *terreus*, and each procedure caused a loss of the TA-biosynthetic ability^7^. Nevertheless, because no intermediates or shunt products were identified, functions of *atG* and *atD* could not be confirmed. Therefore, strains GS-NXAEG and GS-NXAED were constructed, and the heterologous expression results confirmed that AtD but not AtG works in this reaction, in contrast to the previous prediction of AtG function^7^ (Fig. 3A–C). The product was identified as terremutin (**3**, 49.0 mg/L) by LC-MS and ^1^H NMR analysis (Supplementary Fig. S5) and was consistent with the literature data^7^. Moreover, to test whether AtG assists AtD at this step, we then introduced *atG* into strain GS-NXAED to generate GS-NXAED-G transformants, which contain all other intact biosynthetic genes of strain GS-NXAED. Several GS-NXAED-G strains were selected randomly, and none of them produced a higher titer of terremutin (**3**) than GS-NXAED did (Supplementary Table S1). Therefore, AtG did not assist AtD in this reaction. Strains GS-NXAEDC and GS-NXAEGDC were constructed by introducing *atD* and *atC* simultaneously into GS-NXAE and GS-NXAEG, respectively. As compared to GS-NXAED, a specific product was produced by both strain GS-NXAEDC (0.9 mg/L) and strain GS-NXAEGDC (5.8 mg/L) at retention time 25.8 min (Fig. 4A,B,E). The new product had the same retention time and ultraviolet (UV) absorption spectrum as did the TA standard (**1**), and feeding the TA standard into the extracted sample enhanced the compound absorption peak as expected. Besides, LC-MS results indicated m/z of 154 for this compound, in line with TA’s m/z (Supplementary Fig. S6). These results revealed that the newly produced compound was probably the final product: TA. Nonetheless, because production of this newly generated compound was very low in strains GS-NXAEDC and GS-NXAEGDC in shake flask culture, it was not easy to obtain enough of this compound for ^1^H NMR analysis. Consequently, the strain was fermented in a 5 L bioreactor and the target compound was purified. ^1^H NMR data (Supplementary Fig. S6) were in agreement with other results^7^, confirming that AtC catalyzes transformation of terremutin (**3**) into TA (**1**). As shown in Fig. 4B and E, higher TA production was observed in strain GS-NXAEGDC compared with GS-NXAEDC. Nevertheless, given that strains GS-NXAEDC and GS-NXAEGDC were constructed separately, they may contain different biosynthetic gene copies, which affected the concentration of intermediates and final products. Thus, we introduced *atG* into the GS-NXAEDC strain to obtain GS-NXAEDC-G transformants and compared the TA production levels. Three GS-NXAED-G strains were selected for culture randomly and none of them produced a higher titer of TA (**1**) than GS-NXAEDC did (Supplementary Table S2). This finding indicated that AtG neither catalyzed nor assisted AtC in catalyzing the final reaction.

### AtG boosted AtE catalysis

**Figure 5:**
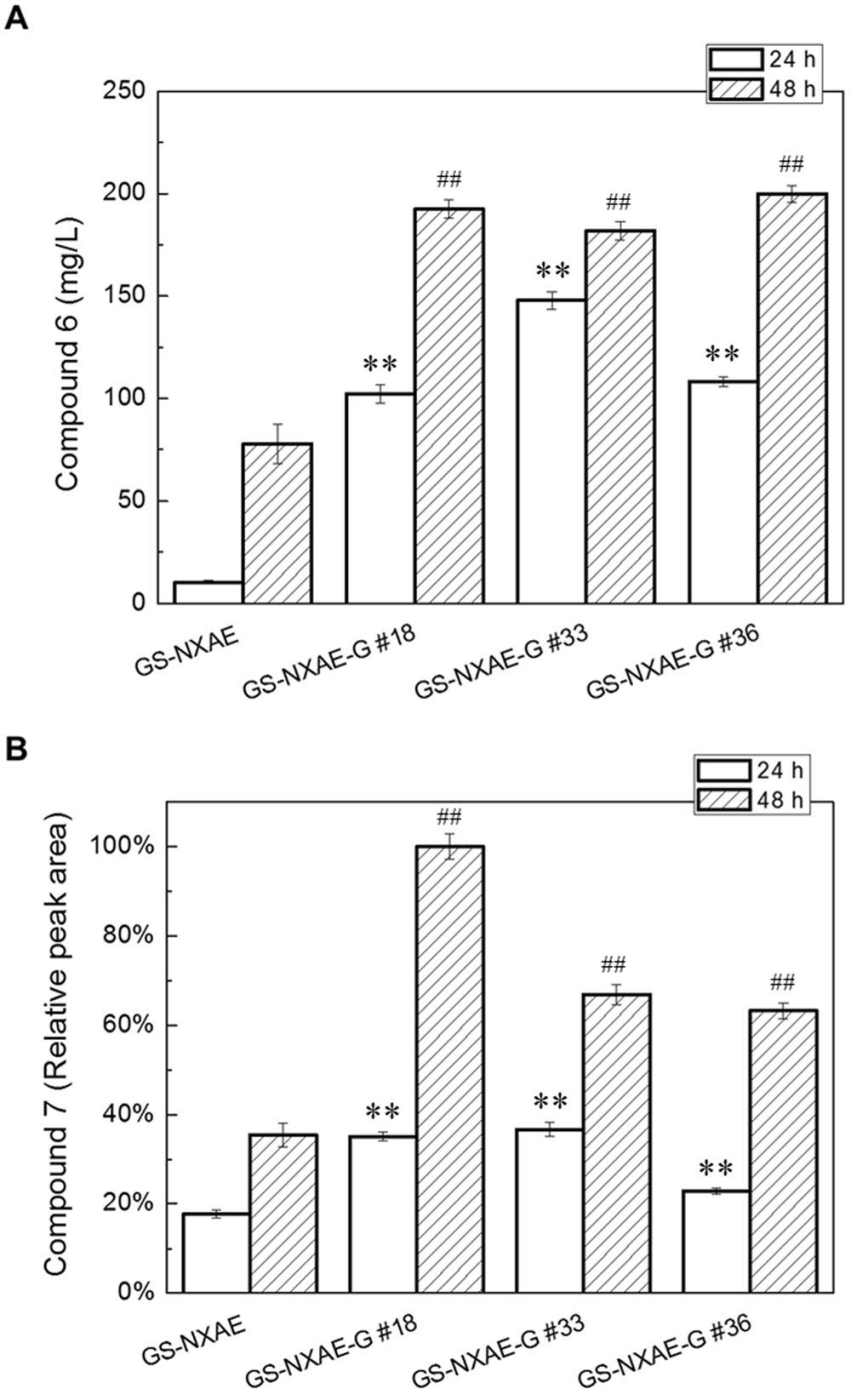
Introduction of atG into strain GS-NXAE improved the biosynthesis of compounds 6 and 7. Three resulting strains (GS-NXAE-G #18, #33, and #36) were selected randomly and tested. Gene copies of *atG* in each strain were not determined, and the production levels of the two compounds differed among the three GS-NXAE-G strains. Compound 6 was quantified properly, but compound 7 was quantified only as the relative HPLC peak area (the highest titer of GS-NXAE-G #18 after 48 h induction was set to 100%). One-way analysis of variance (ANOVA) was employed to determine significant production differences of compounds 6 and 7 between GS-NXAE and each GS-NXAE-G strain. The *P*-value was used to check the significance, and it was significant at *P* < 0.05. OriginPro 8.0 (OriginLab Corporation, USA) was used for ANOVA. **P < 0.01 at 24 h; ^##^*P* < 0.01 at 48 h. Detailed *P*-value for each run was shown in Supplementary Table S8.

**Figure 6:**
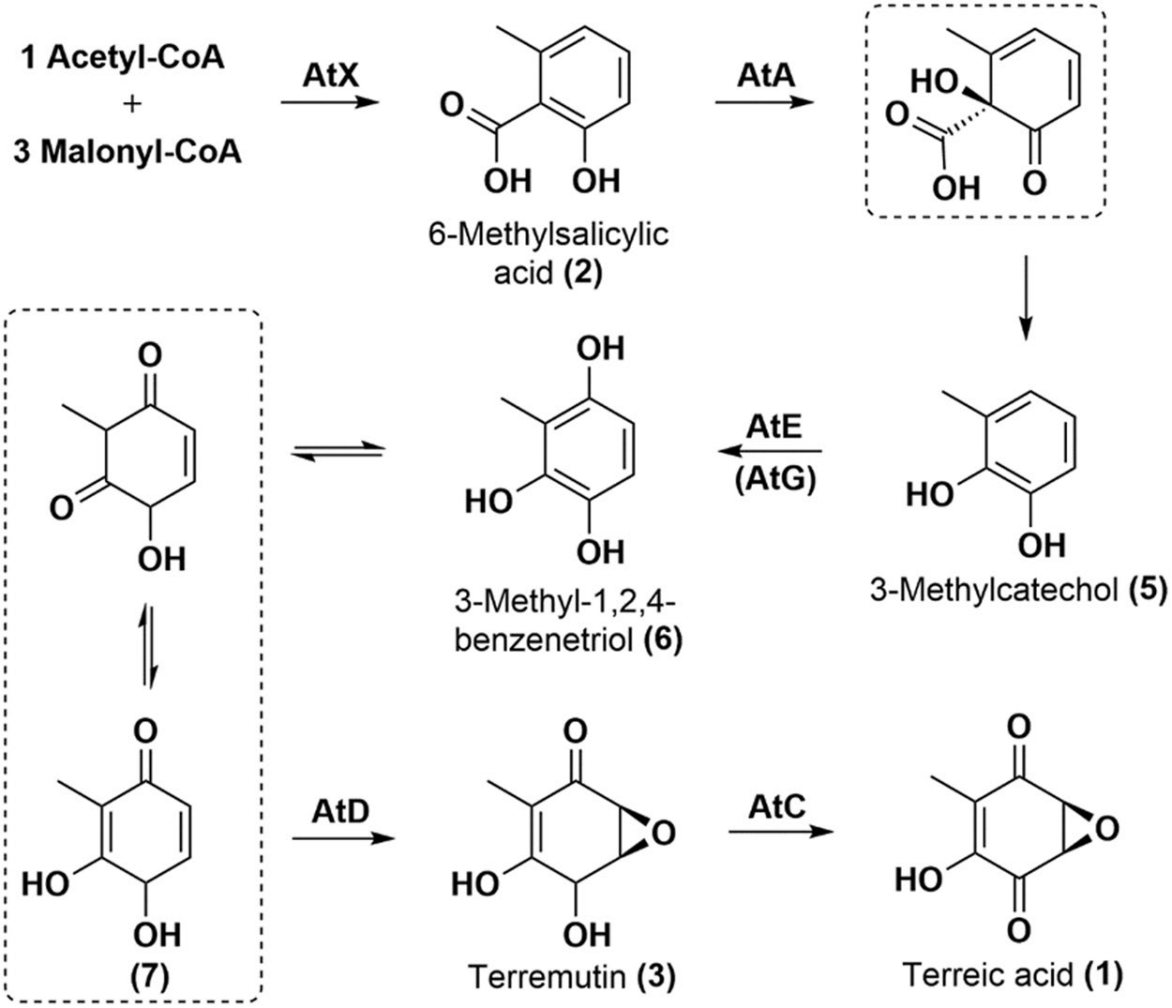
Biosynthetic molecular steps of terreic acid (TA) that were identified by heterologous pathway assembly. Speculated transition compounds are boxed. The AtX (polyketide synthase) catalyzes formation of polyketide 6-MSA **(2)**; AtA (decarboxylase) catalyzes formation of 3-methylcatechol **(5)** by decarboxylative hydroxylation of 6-MSA; AtG (cytochrome P450 monooxygenase) helps AtE (cytochrome P450 monooxygenase) to catalyze formation of 3-methyl-1,2,4-benzenetriol **(6)**; AtD (cyclooxygenase) catalyzes formation of terremutin **(3)** by epoxidation and hydroxyl oxidation of 3-methyl-1,2,4-benzenetriol **(6)**; AtC (GMC oxidoreductase) catalyzes formation of the final product, TA **(1)**, via a reaction of oxidation of the hydroxyl group in terremutin **(3)**.

In the native strain of *A*. *terreus*, accumulation of intermediates or shunt products was not detected after a knockout of *atG*. Additionally, AtG has a putative function of cytochrome P450 monooxygenase, which might work with the other cytochrome P450 monooxygenase: AtE. Accordingly, we introduced *atG* into the GS-NXAE strain to generate GS-NXAE-G transformants. Three strains were selected randomly for analysis of production of compounds **6** and **7**. Both compounds were highly accumulated in GS-NXAE-G strains compared with GS-NXAE, especially **6** after 24 h methanol induction and **7** after 48 h methanol induction (Fig. 5). Levels of **6** were higher even in GS-NXAED-G and GS-NXAEDC-G transformants than in their parent strains GS-NXAED and GS-NXAEDC (Supplementary Table S3). Thus, we may conclude that AtG assists AtE but not AtD or AtC in the TA-biosynthetic pathway. Accordingly, the molecular steps for TA biosynthesis were clarified (Fig. 6), and AtX, AtA, AtE/AtG, AtD, and AtC were found to function stepwise in this process. Nevertheless, how AtG works with AtE to improve the reaction step still kept unknown. To test if AtG interacts with AtE and thereby forms a protein complex, we then conducted a yeast two-hybrid (Y2H) assay^12^ on both proteins. However, the interaction between AtG and AtE was not observed (Supplementary Fig. S7), indicating that they probably not form protein complex and work in other way that needs further deep work to clarify.

## Discussion

To determine the biosynthetic molecular steps for TA (**1**) in *A*. *terreus*, stepwise pathway assembly of TA was performed here in a heterologous host: methylotrophic yeast *P*. *pastoris*. Coding sequences and introns of biosynthetic genes *atA*, *atE*, *atD*, *atG*, and *atC* were identified by reverse transcription, and our findings corrected the results previously submitted to databases. As expected, *P*. *pastoris* expresses AtA, AtE, AtD, AtG, and AtC correctly according to western blotting analysis (Supplementary Fig. S8). Conserved domain analysis indicated that the identified AtA contains a FAD-dependent salicylate 1-monooxygenase domain catalyzing the conversion of salicylate to catechol. Coexpression of *atX* and PPTase-encoding gene *npgA* produced 6-MSA (**2**), and introducing *atA* next led to the biosynthesis of 3-methylcatechol (**5**) by a decarboxylative hydroxylation reaction. Quick BLASTp results on AtA matched several functional uncharacterized salicylate 1-hydroxylases/1-monooxygenases. As reported elsewhere, some salicylate 1-monooxygenase NahG (1-hydroxylating, decarboxylating, EC 1.14.13.1) was identified in *Pseudomonas putida*^13^. A hydrolase OpS4 (UniProtKB accession No. J4VWM7) catalyzing orsellinic acid to 6-methyl-1,2,4-benzenetriol by decarboxylative hydroxylation was also identified in *Beauveria bassiana*^14^. Nevertheless, AtA shares only 34% identity with NahG and 25% identity with OpS4. BLAST results indicates that AtA also shares 32% identity with an *A*. *nidulans* salicylate 1-monooxygenase, SalA, which was characterized biologically but not chemically (Table 1)^15^. Moreover, the oxidation behavior of AtA is similar to that of three other reported FAD-dependent monooxygenases, TropB in tropolone biosynthesis^16^, SorbC in sorbicillinoid biosynthesis^17^, and AzaH in azaphilone biosynthesis^18^, where they perform oxidative dearomatization of their specific substrates. AtA may hydroxylate 6-MSA (**2**) to form an unstable intermediate, which would easily undergo decarboxylation to generate 3-methylcatechol (**5**). For biosynthesis of TA in this case, sorbicillinoids such as sorbicillactones^17^ and azaphilones such as rubropunctatin^18^ require only ring oxidation. Nonetheless, tropolone biosynthesis requires oxidation of both the ring itself and the ring methyl group of a polyketide aldehyde^16^, in contrast to the biosynthesis of citrinin, where only the ring methyl is oxidized^19^. Recently, a 6-MSA decarboxylase, PatG, was identified in patulin biosynthesis and was found to catalyze the first biosynthetic step, namely, decarboxylation but not hydroxylation of 6-MSA to form m-cresol^20^. Thus, these results finally confirmed AtA as a 6-MSA 1-monooxygenase but not the previously predicted 6-MSA decarboxylase^7^. Quick BLASTp of AtE revealed good identity to cytochrome P450 monooxygenases, among which, PatI and PatH (Table 1) in the biosynthesis of patulin have been chemically identified^21^. Introduction of *atE* next led to production of 3-methyl-1,2,4-benzenetriol (**6**) via hydroxylation of 3-methylcatechol, also in agreement with the putative function of cytochrome P450 monooxygenase AtE^7^. Furthermore, **6** easily converted to a specific compound **7** during an active culture phase with decreasing pH, and **7** quickly converted back to **6** after purification, allowing us to infer that it is 3,4-dihydroxy-2-methylcyclohexa-2,5-diene-1-one (**7**), a tautomer of **6** whose chemical structure could not be identified precisely. This uncertainty did not affect the biosynthetic pathway analysis in this case.

The most uncertain molecular step for this biosynthetic pathway is the conversion of 3-methyl-1,2,4-benzenetriol (**6**) to terremutin (**3**) as reported in another work^7^. By experimental gene identification in our study, AtG was confirmed as a protein only 157 aa long. Although AtG contains a conserved cytochrome P450 monooxygenase domain, it shows low identity to other proteins in BLAST results. Particularly, AtG shows much smaller molecular weight than the proteins from BLAST results. Therefore, we predicted that AtG may not work for terremutin production, and introduction of *atG* into one of our strains finally proved this conclusion. By contrast, introduction of *atD* into one of our strains successfully generated terremutin. A conserved-domain analysis in AtD suggested that this protein contains a cupin_2 domain, and Quick BLASTp search yielded ~20 hypothetical homologues with undefined function (from filamentous fungi) with high identity to AtD, including a putative cupin protein (identity of 70%)^22^ and hypothetical dioxygenase PatJ (identity of 62%)^9^ (Table 1). Considering its identified function in this case, it may be designated as an epoxidase with epoxidation functions. On the other hand, AtD showed no significant similarity with some reported epoxidation-mediated epoxidases or cytochrome P450 enzymes, e.g., those participating in the biosynthesis of squalene^23^, lasalocid^24^, mycinamicin^25^, and FD-891^26^. Its enzymatic mechanism and specific biosynthetic roles in other fungi will be an interesting topic for future research. Introduction of *atC* into one of our strains next produced the final product, TA (**1**), proving that AtC functions at this step, in line with the gene knockout results^7^. Conserved-domain analysis indicated that AtC is a GMC oxidoreductase that matches many homologues. Nevertheless, only the versicolorin B synthase (VBS) from *Aspergillus parasiticus*^27,28^ and that from *Aspergillus flavus*^29^ (Table 1) have been characterized.

Fungal cytochrome P450 monooxygenases usually have versatile biocatalytic activities^30,31^. Elsewhere, a knockout of *atA* in native *A*. *terreus* caused potent accumulation of 6-MSA (**2**) whereas a knockout of *atG* did not^7^, meaning that the reaction of 6-MSA (**2**) producing 3-methylcatechol (**5**) is independent of AtG. To further clarify the specific function of *atG*, it was then introduced here into the terremutin-producing or TA-producing strains. These experiments yielded no new compound or improvement of terremutin or TA biosynthesis, suggesting that AtG does not function at these two steps. Another study showed that a knockout of *atE* in native *A*. *terreus* causes strong accumulation of shunt product **4** of 3-methylcatechol (**5**), but a knockout of *atG* does not^7^, meaning that *atE* but not *atG* plays the essential role in this reaction. Our finding that strain GS-NXAE but not GS-NXAG (genes are transcribed and proteins are expressed correctly, Supplementary Figs S8 and S9) produces 3-methyl-1,2,4-benzenetriol (**6**) confirmed this notion. Of note, when *atG* was introduced into the 3-methyl-1,2,4-benzenetriol (**6**)-producing strain (GS-NXAE), it highly improved the biosynthesis of this compound. BLAST searches revealed that AtE and AtG share very low identity. As opposed to AtG, many homologues of AtE (with relatively high identity) were found by Quick BLASTp. Thus, AtG could be a putative cytochrome P450 monooxygenase assisting AtE at the hydroxylation step. Fungal cytochrome P450 usually contains four kinds of conserved motifs^30,31^, and sequence analysis revealed that AtG possesses PER and EXXR motifs whereas AtE contains only PER motifs. To date, a vast number of cytochromes P450 classified into ~400 families have been identified in >2500 fungal species^30–32^. Nonetheless, cooperation of the two types of cytochrome P450 monooxygenase at a single biocatalytic step has seldom been reported. Our protein-protein interaction analysis by Y2H assay preliminarily showed that AtG and AtE did not combine with each other, while how these enzymes (with widely divergent molecular weights) work together at this catalytic step is still an interesting topic for a future study.

After these efforts, we finally clarified the molecular steps in the TA biosynthetic pathway. These data show a fundamental pathway for biosynthesis of TA derivatives, which can be screened for anticancer pharmaceuticals. Moreover, because 6-MSA is an abundant primary intermediate in fungal secondary metabolism^9,20,21,33^, the results we reported in this work may be useful for analysis of the biosynthetic mechanism for other 6-MSA–derived bioactive compounds. In addition, the successful heterologous expression proved that *P*. *pastoris* is a good chassis organism maintaining correct bioactivity of fungal proteins, and these properties certainly facilitate heterologous biosynthesis of fungal secondary metabolites. Moreover, with a short culture phase, clean metabolic background, and easy genetic manipulations^34–37^, this host may be a good choice for either biosynthetic analysis or improvement of production of fungal secondary metabolites.

## Methods

### Strains, plasmids, media, and culture conditions

Genes for TA biosynthesis were cloned from the *at* cluster of *A*. *terreus* NIH2624. *Escherichia coli* TOP10 served as a storage host for plasmids. *P*. *pastoris* GS115 was used as the basic host for heterologous expression of TA-biosynthetic genes. Vectors pAG32 (kindly provided by Prof. Saurabh Joshi in University of California, San Diego)^38^, pPIC3.5 K (Invitrogen), and pPICZ B (Invitrogen) were employed for gene expression. Primers used for identification of introns and construction of expression strains are listed in Supplementary Tables S4 and S5. Plasmids and expression strains in this study are listed in Supplementary Tables S6 and S7. *A*. *terreus* was cultivated at 28 °C in the PDB medium (Hangzhou Microbial Reagent Co., Ltd., China). *E*. *coli* was cultured at 37 °C in the Luria-Bertani (LB) medium consisting of 0.5% yeast extract, 1% tryptone, and 0.5% NaCl. *P*. *pastoris* was cultivated at 30 °C in the YPD medium consisting of 1% yeast extract, 2% tryptone, and 2% glucose for seed preparation, and then cultivated in the minimal medium (MM) composed of 1.34% YNB (Sigma) and methanol for protein expression and compound biosynthesis. Methanol was added to 0.5% (v/v) every 24 h as a carbon source and inducer.

### Molecular biological techniques

For PCR experiments, standard protocols were applied with a PCR amplification kit (TaKaRa, Cat. # R011). Fungal RNA was extracted by means of the RNAsimple Total RNA Kit (TIANGEN Cat. # DP419). Plasmid DNA was isolated from *E*. *coli* using the TIANprep Rapid Mini Plasmid Kit (TIANGEN Cat. # DP105–03). DNA fragments separated in an agarose gel were extracted with the Universal DNA Purification Kit (TIANGEN Cat. # DP214–03). Multiple fragments were assembled via the ClonExpress^TM^ II One Step Cloning Kit (Vazyme Biotech Co., Ltd., China). Strains *P*. *pastoris* GS115 and *E*. *coli* TOP10 and yeast vectors pPICZ B and pPIC3.5 K were purchased from Invitrogen. Transformation of yeast cells and screening of transformants were executed according to *Pichia* protocols^39^. Yeast two-hybrid (Y2H) assay were described in detail in supplementary data file (Supplementary Fig. S7).

### Identification of introns of genes within the *at* cluster

The mRNA sequences of genes within the *at* cluster are already predicted in GenBank (GenBank accession No. CH476602.1), but many of them are different from the prediction results of the SoftBerry software. To confirm the exact positions of introns and express correct enzymes in *P*. *pastoris* for TA biosynthesis, cDNA for each gene was obtained and analyzed by reverse transcription of RNA. An *A*. *terreus* strain was cultivated at 28 °C and 120 rpm in the PDB medium for 7 days, and total RNA was then extracted. A series of primers (Supplementary Table S4) for each gene were used to amplify cDNA of each gene, and the intron positions were then confirmed after DNA sequencing.

### Construction of the GS-NX strain

In our previous study, we successfully implemented 6-MSA biosynthesis in an engineered *P*. *pastoris* carrying *Aspergillus nidulans* PPTase–encoding gene *npgA* and *A*. *terreus* 6-MSAS–encoding gene *atX*^10^. Given that several genes need to be expressed in *P*. *pastoris* and selective markers were limited, *npgA* and *atX* were then inserted into one plasmid in this case. The *npgA* and *atX* expression cassettes with the *AOX1* promoter (P_*AOX1*_) and *AOX1* terminator were amplified from plasmids pPIC3.5K-npgA and pPICZ B-*atX*^10^, respectively. Two pairs of primers TT-AOX-F/TT-HIS4-R and Amp-AOX-F/AOX-TT-R were employed in PCR, and DNA fragment 1 (2353 bp) and fragment 2 (6785 bp) were obtained. Moreover, the selective marker *HIS4* was amplified from plasmid pPIC3.5 K with primers TT-HIS4-F and ori-HIS4-R (fragment 3). Replicon *ori* and a selective marker—ampicillin resistance gene *AmpR* with the *AmpR* promoter—(fragment 4) were amplified together from plasmid pPIC3.5 K with primers HIS4-ori-F and AOX-Amp-R. After that, fragments 1, 2, 3, and 4 were assembled, leading to the expression plasmid pPICβ-npgA-atX. It was transfected into *E*. *coli* TOP10. After PCR verification with primers 5AOX1 and 3AOX1 and DNA sequencing, the correct plasmid was linearized by means of *BspE*I and transfected into wild-type *P*. *pastoris* GS115 by electroporation. The histidine auxotroph was used for screening of transformants for those positive for GS115-NpgA-AtX (GS-NX). The strains were then verified by genotyping PCRs (Supplementary Fig. S10).

### Construction of strains GS-NXA, GS-NXE, GS-NXAE, and GS-NXAG

Genes *atA* and *atE* were obtained by means of primers ZB-atA-F and ZB-atA-his6-R or ZB-atA-F and ZB-atE-his6-R from *A*. *terreus* cDNA, respectively. They were then ligated to the pPICZ B vector digested with *EcoR*I and *Xho*I via seamless assembly, leading to expression plasmids pPICZ B-*atA* and pPICZ B-*atE*. The plasmids were transfected into *E*. *coli* TOP10 and positive transformants with correct plasmids were identified by colony PCR with primers 5AOX1 and 3AOX1 and DNA sequencing. The *GAP* promoter as an integration locus was amplified from *P*. *pastoris* genomic DNA with primers ZB(BglII)-GAP-F and GAP-AOX-R. It was then inserted into pPICZ B-*atA* digested with *Bgl*II to obtain plasmid pPICZ B-*atA*-*GAP*. The *atE* gene containing the promoter and terminator was amplified from pPICZ B-*atE* with primers ZB-BglII-AOX-F and TT-GAP-R, and next inserted into pPICZ B-*atA*-*GAP* digested with *Bgl*II, yielding expression plasmid pPICZ B-*atA*-*GAP*-*atE*. The correct plasmids were then identified by colony PCR with primers 5AOX1 and 3AOX1 and DNA sequencing. After that, pPICZ B-*atA* and pPICZ B-*atE* were linearized with *Pme*I, and pPICZ B-*atA*-*GAP*-*atE* was linearized with *Avr*II, and transfected into strain GS-NX by electroporation. Zeocin at a final concentration of 100 μg/mL served for selection of positive transformants of GS115-NpgA-AtX-AtA (GS-NXA), GS115-NpgA-AtX-AtE (GS115-NXE), and GS115-NpgA-AtX-AtA-AtE (GS-NXAE). For construction of GS115-NpgA-AtX-AtA-AtG (GS-NXAG), the *atG* gene with a flanking sequence was first cloned by means of primers of 3.5k-AOX-atG-F and 3.5K-his6-atG-R. It was then inserted into pPIC3.5 K digested with *EcoR*I and *BamH*I, thereby producing expression plasmid pPIC3.5K-*atG-his6*. The plasmid was next linearized with *Sal*I and transfected into strain GS-NXA by electroporation to generate strain GS115-NpgA-AtX-AtA-AtG (GS-NXAG). The strains were verified by genotyping PCRs (Supplementary Figs S11–S13).

### Construction of strains GS-NXAEG, GS-NXAED, GS-NXAEGD

The *GAP* promoter as an integration locus was amplified from *P*. *pastoris* genomic DNA with primers 3-pGGAP-F and 3-pGGAP-R. After that, it was digested with *Sac*I and *Spe*I and ligated into the same sites of opened vector pAG32 to obtain vector pAGG (Hyg^r^). The *atG* gene was cloned from *A*. *terreus* cDNA using primers ZB-atG-F and ZB-atG-R and inserted into pPICZ B digested with *EcoRI* and *XhoI*. The *atD* gene was cloned from *A*. *terreus* cDNA by means of primers pAG-atD-F and pAG-atD-his6-R and inserted into the pAGG plasmid digested with *Sal*I and *BamH*I. Thus, expression plasmids pPICZ B-*atG* and pAGG-*atD* were obtained. The correct plasmids were then identified by colony PCR with primers 5AOX1 and 3AOX1 and DNA sequencing. Considering the selection marker and His tag, the *atG* containing a promoter and terminator was amplified from pPICZ B-*atG* with primers 3,5k-AOX-atG-F and 3.5k-his6-atG-R, and next inserted into vector pPIC3.5 K digested with *EcoR*I and *BamH*I, so that pPIC3.5K-atG carrying a His tag was constructed. The *atG* gene containing a promoter and terminator was amplified from pPICZ B-*atG* with primers ZB-BglII-AOX-F and TT-AOX-R, and inserted into pPICZ B-*atA*-*GAP*-*atE* digested with *Bgl*II, leading to expression plasmid pPICZ B-*atA-GAP-atE-atG*. The *atD* gene carrying the promoter and terminator was amplified from plasmid pAGG-*atD*, followed by insertion into pPICZ B-*atA-GAP-atE-atG* digested with *Bgl*II, leading to expression plasmid pPICZ B-*atA-GAP-atE-atG-atD*. The correct plasmids were identified by colony PCRs and DNA sequencing. After that, plasmids pAGG-*atD*, pPICZ B-*atA-GAP-atE-atG*, and pPICZ B-*atA-GAP-atE-atG-atD* were linearized by *Avr*II digestion and transfected into strains GS-NXAE, GS-NX, and GS-NX by electroporation, separately. Zeocin (100 μg/mL) and hygromycin (750 μg/mL) served for screening of transformants for those positive for GS115-NpgA-AtX-AtA-AtE-AtD (strain GS-NXAED), GS115-NpgA-AtX-AtA-AtE-AtG (strain GS-NXAEG), or GS115-NpgA-AtX-AtA-AtE-AtG-AtD (strain GS-NXAEGD). These strains were then verified by genotyping PCRs (Supplementary Figs S14–S16).

### Construction of strains GS-NXAEDC and GS-NXAEGDC

The *atC* gene was cloned from *A*. *terreus* cDNA via primers ZB-atC-F and ZB-atC-his6-R and inserted into pPICZ B digested with *EcoR*I and *Xho*I, leading to expression plasmid pPICZ B-*atC*. The correct plasmid was then identified by colony PCR with primers 5AOX1 and 3AOX1 and DNA sequencing. Genes *atD* and *atC* containing a promoter and terminator were amplified from pPICZ B-*atG* and pAGG-*atD* with primers pAG-AOX-F and AOX-TT-R or TT-AOX-F and pAG-TT-R, respectively, and then inserted into pAGG digested with *Sal*I and *BamH*I, thus generating expression plasmid pAGG-*atD-atC* finally. The correct plasmids were identified by colony PCR. Next, the pAGG-*atD-atC* vector was linearized by *Avr*II digestion and transfected into strains GS-NXAE and GS-NXAEG. Hygromycin at a final concentration of 750 μg/mL was used for screening of transformants for those positive for GS115-NpgA-AtX-AtA-AtE-AtD-AtC (strain GS-NXAEDC) and GS115-NpgA-AtX-AtA-AtE-AtG-AtD-AtC (strain GS-NXAEGDC). These strains were verified by genotyping PCRs (Supplementary Figs S17 and S18).

### Creation of strains GS-NXAE-G, GS-NXAED-G, GS-NXAEDC-G

To eliminate the influence of biosynthetic copies on compound production levels and to test whether *atG* can assist the functioning of *atD*, *atC*, or *atE*, vector pPIC3.5K-*atG* linearized with *Nco*I was transfected into strains GS-NXAE, GS-NXAED, and GS-NXAEDC by electroporation. G418 at a final concentration of 0.25 mg/mL served for screening of transformants for those positive for GS-NXAE-G, GS-NXAED-G, and GS-NXAEDC-G genotypes. These strains were verified by genotyping PCRs in the same way as GS-NXAE, GS-NXAED, and GS-NXAEDC were. Additionally, vector pPIC3.5K-*atG* was separately integrated at the *HIS4* site and verified with primers 5′AOX and atG-yz-R as well as atG-yz-F and 3′AOX.

### Transcriptional analysis

Total RNA was extracted according to *Pichia* protocols^39^. RQ1 RNase-Free DNase (Promega) was employed to remove the residual DNA. Reverse transcription was conducted by means of the PrimeScript^TM^ RT Reagent Kit (TaKaRa). Wild-type *P*. *pastoris* GS115 served as a negative control.

### Protein expression and western blot analysis

Strains GS-NXA and GS-NXAE carry AtA and AtE with a His tag. Plasmids pAGG-*atD*, pPIC3.5K-*atG*, and pPICZ B-*atC* were engineered to contain a His tag and then linearizd and transfected into the wild-type *P*. *pastoris* GS115 by electroporation to obtain strains GS115-AtD-HIS_6_, GS115-AtG-HIS_6_, GS115-AtC-HIS_6_. These strains were then analyzed to test whether the TA biosynthetic enzymes could be correctly expressed in *P*. *pastoris*. After induction with 0.5% methanol in the YNB medium for 48 h, 30 OD_600_ units of yeast cells were harvested by centrifugation (3000 × *g*, 5 min) and washed twice with precooling 50 mM potassium phosphate buffer (pH 7.0), and then resuspended in 1 mL of binding buffer (50 mmol/L K_3_PO_4_, pH 7.0, 1 mmol/L phenylmethylsulfonyl fluoride [PMSF]). The suspension was added into a 2.0 mL screw cap tube with 1 g of zirconium followed by disruption in a BeadBeater (Minilys, Bertin Technologies) for 8 cycles (30 s vibration and 1 min of an ice bath in each cycle). The lysate was centrifuged (12000 × *g*, 30 min), and the precipitate was discarded. For western blot analysis, 20 μL of total protein samples (analyzed with the Bradford protein assay kit, Tiangen Biotech) were loaded into polyacrylamide gel wells and separated under denaturing conditions^40^. After that, the proteins were transferred onto a polyvinylidene difluoride (PVDF) membrane. The mouse anti-His antibody (Tiangen Biotech) and the peroxidase-conjugated goat anti-mouse immunoglobulin G (Tiangen Biotech) served as the primary antibody and secondary antibody, respectively.

### Extraction and identification of TA and intermediates

After centrifugation for 5 min at 3000 × *g*, 50 mL of the supernatant was extracted with an equal volume of ethyl acetate. The organic phase was removed in a rotary evaporator at 40 °C, and the remainder was dissolved in 1 mL of methanol. Further analysis of the extracts was carried out by HPLC on a C_18_ column (Kromasil^TM^, Sweden, 250 mm × 4.6 mm × 5 μm, 100 Å spherical silica) at a flow rate at 0.4 mL/min and detection by UV absorbance at 254 nm (intermediates) and 330 nm (TA). The gradient system was 0.1% acetic acid in H_2_O (solvent A) and acetonitrile (solvent B). Gradient conditions were as follows: minute 0, 10% B; minute 40, 30% B; minute 50, 85% B; minutes 50–55, 100% B (for terreic acid); or minute 0, 15% B; minute 40, 85% B; minutes 40–45, 100% B (for other compounds); or minute 0, 15% B; minute 30, 85% B; minutes 30–35, 100% B (for other compounds). To confirm the compounds, further analysis was performed by LC with high-resolution MS (LC-HRMS; Agilent 6230 TOF LC-MS) and NMR (Bruker-AM-400-spectro) in a freeze-dried sample dissolved in deuterated DMSO or deuterochloroform for ^1^H NMR, ^13^C NMR, HMBC, and HSQC analyses.

### Data availability

All data generated or analysed during this study are included in this published article (and its Supplementary Information files). Genes re-annotated are also deposited in GenBank and the assigned accession numbers are provided in this published article.

### Ethical approval and informed consent

We declare that this paper does not report any data collected from humans or animals.

## Electronic supplementary material

Supplementary information
