# Supplementary material for: Heterologous pathway assembly reveals molecular steps of fungal terreic acid biosynthesis
Source: Sci Rep. 2018 Feb 1;8:2116. doi: 10.1038/s41598-018-20514-x (PMC5794859; doi:10.1038/s41598-018-20514-x)
Supplement: Supplementary file 1 — Supplementary information [file 41598_2018_20514_MOESM1_ESM.pdf]

Supplementary Information

Heterologous pathway assembly reveals molecular steps of fungal terreic acid biosynthesis

Chuixing Kong[1], Hezhou Huang[1], Ying Xue[1], Yiqi Liu[1], Qiangqiang Peng[1], Qi Liu[1], Qin Xu[1], Qiaoyun Zhu[1], Ying Yin[1], Xiangshan Zhou[1], Yuanxing Zhang[1,2], Menghao Cai[1,*]

[1]State Key Laboratory of Bioreactor Engineering, East China University of Science and Technology, 130 Meilong Road, Shanghai 200237, China

[2]Shanghai Collaborative Innovation Center for Biomanufacturing, 130 Meilong Road, Shanghai 200237, China

*Corresponding author. *Tel./fax:* +86-21-64253306. *E-mail address:* cmh022199@ecust.edu.cn

Strains	Titre (mg/L)	
	**Methanol induction for 24 h**	**Methanol induction for 48 h**
GS-NXAED	17.8	49.0
GS-NXAED-G #1	15.1	40.8
GS-NXAED-G #2	14.8	34.1
GS-NXAED-G #3	16.3	39.5
GS-NXAED-G #6	17.2	42.4

Supplementary Table S1. Titres of terremutin (compound 3) produced by the strain GS-NXAED and its *atG* co-expression transformants of GS-NXAED-G

Strains	Titre (mg/L)	
	**Methanol induction for 24 h**	**Methanol induction for 48 h**
GS-NXAEDC	5.7	5.3
GS-NXAEDC-G 14#	4.9	4.7
GS-NXAEDC-G 43#	4.0	4.0
GS-NXAEDC-G 56#	4.7	4.2

Supplementary Table S2. Titres of terreic acid (compound 1) produced by the strain GS-NXAEDC and its *atG* co-expression transformants of GS-NXAEDC-G

Strains	Titre	
	**Compound 6 (mg/L)**	**Compound 7 (peak area)**
GS-NXAED	47.2	14014
GS-NXAED-G 1#	75.2	11934
GS-NXAED-G 2#	62.3	8406
GS-NXAED-G 3#	95.4	11471
GS-NXAED-G 6#	71.4	12043
**Strains**	**Titre**	
	**Compound 6 (mg/L)**	**Compound 7 (peak area)**
GS-NXAEDC	3.8	3543
GS-NXAEDC-G 14#	10.2	3514
GS-NXAEDC-G 43#	14.9	1925
GS-NXAEDC-G 56#	20.0	1085

Supplementary Table S3. Titres of compound 6 and 7 produced by the strains GS-NXAED and GS-NXAEDC after 48 h methanol induction comparing with their *atG* co-expression transformants of GS-NXAED-G and GS-NXAEDC-G

Primer	Sequence (5' to 3')[a]
atA-F	CGGGCTTGCTCCCTTCA
atA-R	GAGTGGACGCCCGAAT
ZB-atA-F	TTATTCGAAACGAGGATGACTTTCTCTAATCGGGTTT
ZB-atA-his6-R	GGCGGCCGCCGCGGCTCAATGATGATGATGATGATGAACAGTAGATGTTGCCACA
atC-F	TCCATACTGGGACTCATTCA
atC-R	AGCCCATCACCTCAGAAA
ZB-atC-F	TTATTCGAAACGAGGATGCGCGTGTTTCCC
ZB-atC-his6-R	GGCGGCCGCCGCGGCTCAATGATGATGATGATGATGCTTACTCAAGATATCCTCTGCA
atD-F	ATGGGTCATGGATGGT
atD-R	CACTCAATCCATTTGGCTAT
ZB-atD-F	ACTAATTATTCGAAACGAGGATGGGTGAACTGTTCAAGT
ZB-atD-R	TCTGAGATGAGTTTTTGTTCGGAGCGCTTTCGGAGC
atD-yz-F	TTTTATTCAGACCCTTTGC
atD-yz-R	CACCACGCACATCCAGTA
pAGG-5AOX-F	GCTTCGTACGCTGCAGGTCGACAGATCTAACATCCAAAGACGAA
pAGG-TT-R	GCCTTAATTAACCCGGGGATCCGGATCCGCACAAACGAAG
atE-1F	CTGAATTAAGCCACAATAGACA
atE-2F	GCAATACGGCGGTATCTT
atE-R	TCAATCTTCCACCCAACTC
ZB-atE-F	ACTAATTATTCGAAACGAGGATGGCAACTCTTCCCATT
ZB-atE-his6-R	GGCGGCCGCCGCGGCTCAATGATGATGATGATGATGATCATACTTGGCAAAAACA
atG-1F	CGTACGAGGTCATGTCT
atG-2F	ATGCTCCTTGCTGC
atG-3F	AGACCTCGTCAATAATGTCA
atG-4F	GCTCCGTGAAATTCATGT
atG-5F	TCTAAGCGAGAAACGGT
atG-6F	GCCTACACTAGACCAGG
atG-R	TGCCGTCACAGTTTGG
ZB-atG-F	TTATTCGAAACGAGGATGATCTCATCCGTTCCA
ZB-atG-R	GGCGGCCGCCGCGGCCTATTGCACCGTCTTGC
3.5k-AOX-atG-F	AAAACAACTAATTATTCGAAATGATCTCATCCGTTCCA
3.5K-his6-atG-R	GCGAATTAATTCGCGGCCGCCTAATGATGATGATGATGATGTTGCACCGTCTTGCG
5'AOX	GACTGGTTCCAATTGACAAGC
3'AOX	GGCAAATGGCATTCTGACAT

Supplementary Table S4. Primers used introns identification. [a] Relevant restriction sites are underlined.

Primer	Sequence (5' to 3')[a]
Amp-AOX-F	TGTTGAATACTCATTGAGATCAGATCTAACATCCAA
Amp-AOX-R	TTAGATCTGATCTCAATGAGTATTCAACATTTCCG
AOX-TT-R	TTGGATGTTAGATCTGGATCCGCACAAACGAA
TT-AOX-F	CGTTTGTGCGGATCCAGATCTAACATCCAAAGACGA
TT-HIS4-R	CTATGGTGTGTGGGGGATCCGCACAAACGAA
TT-HIS4-F	TCCCCCACACACCATAGATGACATTTCCCTTGCTACCT
HIS4-ori-F	CGATAGATCTAACCGTGTGAGCAAAAGGCCAGCAA
ori-HIS4-R	GGCCTTTTGCTCACACGGTTAGATCTATCGAATC
atX-yz-R	ATCCCGAGCAACCATACT
atX-yz-F	GCGGAGGGCAGAAATAGA
AOX-F	AACATCCAAAGACGAAAG
TT-R	CTGTACTCTGAAGAGGAGT
Amp-F	ATGAGTATTCAACATTTC
Amp-R	TTACCAATGCTTAATCAGTGA
His-F	ATGACATTTCCCTTGCTA
His-R	TTAAATAAGTCCCAGTTTCT
npgA-F	ATGGTACATCGACACCCGCC
npgA-R	CGAATTAATTCGCGGCCGCT
ZB-GAP-F	TTGGTCATGAGATC<u>AGATCT</u>TTTTTGTAGAAATGTCTT
GAP-AOX-R	TCGTCTTTGGATGTTATAGTTGTTCAATTGATTGAAATAG
ZB-AOX-F	TTGGTCATGAGATC<u>AGATCT</u>AACATCCAAAGACGAA
TT-GAP-R	ACATTTCTACAAAAA<u>GGATCC</u>GCACAAACGAA
atA-f	CATTTGCTCGGAGAAAGC
atA-r	ATCCCTAGCAAAGTCATTC
atE-f	ACTCTGTGATCGGCACG
atE-r	TCTTGGCGCGGATTCCG
atX-F'	GCGGAGGGCAGAAATAGA
atX-R'	ATCCCGAGCAACCATACT
atA-F'	TCGGCAAGCGACTCAT
atA-R'	CCACATAGCGGTCGTTG
atE-F'	GCAATACGGCGGTATCTT
atE-R'	CCACGGCGATAAACAGA
ZB-AOX-F	TTGGTCATGAGATC<u>AGATCT</u>AACATCCAAAGACGAA
TT-AOX-R	TCGTCTTTGGATGTT<u>GGATCC</u>GCACAAACGAAG
atA-yz-F	TCGGCAAGCGACTCAT
atA-yz-R	CCACATAGCGGTCGTTG
atE-yz-F	GAGAACCGACGAAAGGC
atE-yz-R	CCCTCCATCAATGTGCC
atG-yz-F	GGGAAAGACAGAGTATTGGC
atG-yz-R	CGTCTTGCGGACTTTCA
atD-yz-F	TTTTATTCAGACCCTTTGC
atD-yz-R	CACCACGCACATCCAGTA

atC-yz-F	GCCACTTATCCTTATGATGC
atC-yz-R	CGGTGCGAAGCCATCT
TT-pPAG-5AOX-F	TGCGGATCCGGATCCCCGGGAGATCTAACATCCAAAGA
TT-(PacI)pPAG-R	ATCTGGCGCGCCTTAATTAAGGATCCGCACAAACGA
atG-F’	GATCTCATCCGTTCCATATCG
atG-R’	GACTTTCACTTGAATACCAGG
atD-F’	TGATGCGACAAACCTACC
atD-R’	TCCTCCCCATTCTCCTTC
atC-F’	CATACATTGCTGTCTCGG
atC-R’	CTTCGGGGAAATCATCTG
AD-atG-F	GCCATGGAGGCCAGTGAATTCATGATCTCATCCGTTCCATATCG
AD-atG-R	CAGCTCGAGCTCGATGGATCCCTAATGATGATGATGATGATGTTGCA
BK-atE-F	GAGGAGGACCTGCATATGGCCATGGCAACTCTTCCCATTCT
BK-atE-R	AGGGGTTATGCTAGTTATGCGGCCTCAATGATGATGATGATGATGATCATACT

Supplementary Table S5. Primers used for construction and verification of expression strains. [a] Relevant restriction sites are underlined.

**Plasmid**	**Characteristic(s)**	**Source or reference**
pUC18	Ampicillin^R; *E. coli* subcloning vector	Invitrogen
pPIC3.5K	Ampicillin^R G418^R; P_{AOX1}-based expression vector	Invitrogen
pPICZ B	Zeocin^R; P_{AOX1}-based expression vector	Invitrogen
pAGG	Ampicillin^R Hygromycin^R; P_{GAP}-based expression vector	This study
pPIC3.5K-*npgA*	pPIC3.5K derivative carrying P_{AOX1}-*npgA* expression cassette	This study
pPICZ B-*atX*	pPICZ B derivative carrying P_{AOX1}-*atX* expression cassette	This study
pPICβ-*npgA*-*atX*	pPIC3.5K derivative carrying P_{AOX1}-*atX* and P_{AOX1}-*npgA* expression cassette	This study
pPICZ B-*atA*	pPICZ B derivative carrying P_{AOX1}-*atA-His tag* expression cassette	This study
pPICZ B-*atE*	pPICZ B derivative carrying P_{AOX1}-*atE-His tag* expression cassette	This study
pPICZ B-*atA-GAP* -*atE*	pPICZ B derivative carrying P_{AOX1}-*atX* and P_{AOX1}-*atE* expression cassette and *GAP* integration locus	This study
pPICZ B-*atG*	pPICZ B derivative carrying P_{AOX1}-*atG* expression cassette	This study
pAGG-*atD*	pAGG derivative carrying P_{AOX1}-*atD* expression cassette	This study
pPICZ B-*atA* -*GAP*-*atE*-*atG*	pPICZ B derivative carrying P_{AOX1}-*atX*, P_{AOX1}-*atE* and P_{AOX1}-*atG* expression cassette and *GAP* integration locus	This study
pPICZ B-*atA*-*GAP* -*atE*-*atG*-*atD*	pPICZ B derivative carrying P_{AOX1}-*atX*, P_{AOX1}-*atE*, P_{AOX1}-*atG* and P_{AOX1}-*atD* expression cassette and *GAP* integration locus	This study
pAGG-*atD*-*atC*	pAGG derivative carrying P_{AOX1}-*atD* and P_{AOX1}-*atC* expression cassette	This study
pPIC3.5K-*atG*	pPIC3.5K derivative carrying P_{AOX1}-*atG* expression cassette	This study
pGADT7	Ampicillin^R, *LEU2*, P_{ADH1} based expression vector with activation domain (AD)of GAL4	Clontech
pGBKT7	Kanamycin^R, *TRP1*, P_{ADH1} based expression vector with BD domain of GAL4	Clontech
pGADT7-53	pGBKT7 derivative containing P_{ADH1}-GAL4AD-(murine P53protein) (73-390 AA) expression cassette vector	Clontech
pGBKT7-T	pGADT7 derivative containing P_{ADH1}-GAL4BD-(SV40 large T antigen) (87-708 AA) expression cassette	Clontech
pGADT7-*atG*	Ampicillin^R; pGADT7 expressing *atG*	This study
pGBKT7-*atE*	Kanamycin^R; pGBKT7 expressing *atE*	This study

Supplementary Table S6. Plasmids used in this study. ^{R} resistance to indicated antimicrobial agent.

Strain	Genotype	Source
E. coli		
Top 10	F'[*lacI*^{q} Tn*10*(Tet^{r})] *mcrA* Φ80*lacZ* ΔM15 Δ*lac X74 deoR recA1*	Invitrogen
P. pastoris		
GS115 (Wild-type)	*his4*	Invitrogen
GS-NX	GS115 *his4*::pPICβ-*npgA-atX* (*HIS4*)	This study
GS-NXA	GS115 *his4*::pPICβ-*npgA-atX* (*HIS4*) *AOX*::pPICZ B-*atA* (*Sh ble*)	This study
GS-NXE	GS115 *his4*::pPICβ-*npgA-atX* (*HIS4*) *AOX*::pPICZ B-*atE* (*Sh ble*)	This study
GS-NXAE	GS115 P_{GAP}::pPICZ B-*atA-GAP-atE* (*HIS4, Sh ble*)	This study
GS-NXAEG	GS115 P_{GAP}::pPICZ B-*atA-GAP-atE-atG* (*HIS4, Sh ble*)	This study
GS-NXAED	GS115 P_{GAP}::pPICZ B-*atA-GAP-atE* (*HIS4, Sh ble*)::pAGG-*atD* (*Hyg*)	This study
GS-NXAEGD	GS115 P_{GAP}::pPICZ B-*atA-GAP-atE-atG-atD* (*HIS4, Sh ble*)	This study
GS-NXAEDC	GS115 P_{GAP}::pPICZ B-*atA-GAP-atE* (*HIS4, Sh ble*)::pAGG-*atD-atC* (*Hyg*)	This study
GS-NXAEGDC	GS115 P_{GAP}::pPICZ B-*atA-GAP-atE-atG* (*HIS4, Sh ble*)::pAGG-*atD-atC* (*Hyg*)	This study
GS-NXAE-G	GS115 P_{GAP}::pPICZ B-*atA-GAP-atE* (*HIS4, Sh ble*):: pPIC3.5K-*atG* (*HIS4*)	This study
GS-NXAED-G	GS115 P_{GAP}::pPICZ B-*atA-GAP-atE* (*HIS4, Sh ble*) :: pAGG-*atD* (*Hyg*)::pPIC3.5K-*atG* (*HIS4*)	This study
GS-NXAEDC-G	GS115 P_{GAP}::pPICZ B-*atA-GAP-atE* (*HIS4, Sh ble*)::pAGG-*atD-atC* (*Hyg*)::pPIC3.5K-*atG* (*HIS4*)	This study
Saccharomyces cerevisiae for Y2H assay		
Y2H Gold	*abar, his3, ade2, mel1*	Clontech
Sc-BD-T/AD-53	Y2H Gold transformed with plasmids pGADT7-53 and pGBKT7-T	This study
Sc-BD/AD	Y2H Gold transformed with plasmids pGADT7 and pGBKT7	This study
Sc-BD/AD-AtG	Y2H Gold transformed with plasmids pGADT7-AtG and pGBKT7	This study
Sc-BD-AtE/AD	Y2H Gold transformed with plasmids pGADT7 and pGBKT7-AtE	This study
Sc-BD-AtE/AD-AtG	Y2H Gold transformed with plasmids pGADT7-AtG and pGBKT7-AtE	This study

Supplementary Table S7. Strains used in this study.

One-way ANOVA (*P value*)	GS-NXAE			
	Compound 6		Compound 7	
	24 h	48 h	24 h	48 h
**GS-NXAE-G #18**	2.66E-7	1.94E-6	2.17E-5	8.61E-6
**GS-NXAE-G #33**	4.58E-8	2.79E-6	4.78E-5	9.67E-5
**GS-NXAE-G #36**	2.01E-8	1.47E-6	1.48E-3	1.09E-4

Supplementary Table S8. ***P*-values by one-way analysis of variance (ANOVA) for determining significant production differences of compounds 6 and 7 between GS-NXAE and each GS-NXAE-G strain. It was significant at $P < 0.05$.**

***A. terreus* NIH2624 *atA* (GenBank accession No. KY950680)**

ATGACTTTCTCTAATCGGGTTTCGGTTGCCATCATTGGAGGTGGTATCGGTGGGCTCAGCCTCGCCATCG
GCCTGCTTCAGAACAAAAACCTGGATGTCGCCATCTACGAAACCGCACCCAAGTTCGCCGAAATCGGGGC
CGGCGTTGCCTTGGGCCCAAATGCCCAACACGCTCTCGCCCTGATTTCCCCCGCCGCCGAGCACGCATTT
CGAATCCACGCAACGACTAGTCTCTCGCCGGAATTCGAACACGTCTGGTTTGATTTCCGGAACGGCAACG
CCGGGGAAAAAGACGGTGAGGTCCTCAGCAAAGTCGAGAATGAGACCGGCCAGCAAACGGTCCATCGGGC
GAAATTCCTCGACGAGCTCGTTAAGCTCATTCCGCGGGAAATCGCCCACTTCGGCAAGCGACTCATCCAC
ATCCAGAAAGATCCTGTGTCCGGGGGTGCTCAGTACAAGCTCTTCTTTGAAGATGGAACGACGGCCTCTG
CCGACTGTGTCATTGGCGCTGATGGAATCCACAGTTCGGTCAGGAAGCATTTGCTCGGAGAAAGCCATCC
TGCTGCCACCCCAGTGTTCACCGGAACTGTGGTTTATCGTGGCCTTATACCGATGGATGTCGCCCGCGAC
GCCATTGGCGAATTTGCCGATAATTCGTATATGTGGTGCGGGGACGGGGGCATGGTCATGACCTATCCCA
TTGATCACGGCGAGACCCTGAATGTGGTTGGAACCCGAAATGACAAGGGAAGATGGGACGGTCCCCCCTA
CACCAGGCCTGTGGACGAGGAAACAGTGCGGAACGATTTTATGGGCTGGGGAGAGATCCCTAGCAAAGTC
ATTCAGGTATAACTCAGCTGACCCTATTTACCGGCCAGACTTAGTTAACTACCATGTTGAATATATCTAG
CTCCTGAAACAGCCAACGATGTGGGCCATCCTGGACCATTATCCAGCGCCCTACTACTATTCCGGCAACG
TCGCGATAATGGGGGACGCCGCACATGCAACCACTCCGTTCCAGGGTGCTGGTGCGGGGCAGGCAATCGA
GGATGCATTGGTCTTATCGACCCTCTTTCAGCGGGTCACCCACTCCGGACTGGTGAGGCCTGCCTTGGCC
GCGTACAACAATGTCAGACTACGCCGGACTCAGAAGGTTGTCGCCACCAGTCGCGATGCACTGAGATTGT
TCTGTTTCAACGACCGCTATGTGGACGGAGACGCGCAGCGCTGGAGAGAGGTGTGGAACGGCCGCATGGA
TTGGCTATGGGGAATGGATTTGGAGAAACAGAACAGGGATGCAGTCAACCTTTTTGCGGACATTGTTGAG
AAGCAATCGTCTGCTAGGGAGACAATGCCCAAATTTGGGCCCGTCCCCTTCCCTGTGGCAACATCTACTG
TTTGA

***A. terreus* NIH2624 *atE* (GenBank accession No. KY950681)**

ATGGCAACTCTTCCCATTCTGGACCTGTGGTCCACCAACAAGGCCCTCGTATCCTTGACCATAAGCGCAAGTCTT
CTGCTCGTCTCACTTCTCGTGTCCTTGTTCCAGGTGGGACGTCGGGAACGCGGTCTTCCGCCAGGTTAGCTCAT
GGGCCCCAGAATACAGACCAAACGCACTGGCTGAAACAGCAGAAAAAATTGCAGGCCCTCCGACTCGACTCG
TGCTGGGAAATGAACACGAGATTCCGAAAGCTGATAGCCACTTCCTGTATGCAAGGCACCGCATCTTTCGTGCG
TGAATCGCGTCTAACCATGCTCTCTTTGTGCAGGATGTCCAAATGGGCCAAGCAATACGGCGGTATCTTTTCTCT
GAAACGCTTCCGGAACACCACCATCGTTTTGACGGACTGGAAGATCATGAAGGAGCTGGTTGACAAGAAAAGC
ACCAACTTCAGCCACCGCCCCCCGTCCAAGGTAGCCGATCTCATCACTCGCGGCAACCACATCCTCATGATGCA
ATATGGCGAGACATGGCGGACAATGCGCAAGCTCATCCACCAGTACCTCATGGAATCCCAGTGCGAGAAGGAG
CACTGGAAGGTCCAGGAGGCGGAGGCCGCCCAGATGCTGCATGATTTTCTGGTTGACCCAGAGAACCATATGA
AGCATCCTAAGCGGTACAGCAACAGCATTACCATGTCTTTAGGTACGTTGCGTTCATCCCTTTTTCTAGCTCCTCG
CCATCTGCAGCTGCTGAGCTGGATATGCAGTCTTCGGAATCCGCGCCAAGAGCGTCAATGACGAATACATGACC
CGGCTGTACAGCCTCATGGAGAAGTGGTCTCTGGTGCTCGAGACCGGCGCCACGCCTCCGGTTGACTCCTGGC
CACTCCTCCAATGGATCCCTGAGAGATTCATGGGCTACTGGCGGCGACGGGCGACCGAGGTGGGCGACCTGAT
GACGGGTCTGTACACGGAGGTGCTGCATGTCATCGAGAACCGACGAAAGGCGGGCATCTACAAGGACTCCCTC
ATGGACCGGGTGCTGGACAAGAAAGACAAGTACCGATTCGACGAGCACCAACTAGCATTCCTCGGAGGCACAT
TGATGGAGGGAGGATCAGATACATCTTCGTCACTGATCTTGGCCATTGTGCAGGCGATGACTCAATACCCTGAG
GTTCAGAAGAAGTATGTCCCCAATTTCCATATCCCATTTCCGTTACTGATATAGACCCGTGTAGAGCACACGCTG
AAATCGACTCTGTGATCGGCACGGATCGGTCCCCGGCCTGGTCCGACTTCCGCAAGCTGCCTTACATCAATATG
ATGATCAAAGAGGCGCACCGCTGGCGGCCAGTCCTGCCACTGGGGGTCGTCCATGGGCTAGCCACAGACGACT
CGTACAATGGGATGCATCTCCCGAAGCACTCTACCGTGATCCTCAACGTGTGGGGCATGCACATGGACCCGGAC
CGATTCGAAAATCCCGACGCCTTCATTCCGGAACGCTATGCCAACTTTCCGGAGCTGGCGCCTCACTATGCCGC
ACTCGCAGACGGTGCGGCGCGAGACCACTTCGGCTATGGCGCAGGCCGTCGTATCTGTCCCGGCATCCACCTTG
CGGAACGGAATCTGTTTATCGCCGTGGCGAAGCTGCTGTGGGCGTTTGAGTTCAAGAACAACCCCGCCGGGAA
GAATGATGCGAGCGCGGAGACTGGGAGCAGCCAGGGATTCATGCATTGTGTAAAGGACTACGACGCGATCGTC
ACGGTCCGGGGGGAGGAGAGAAGACAGACTATTCTCAGGGAATTGGAGCAGGCGCAGACTGTTTTTGCCAAG
TATGATTGA

***A. terreus* NIH2624 *atG* (GenBank accession No. KY950683)**

ATGATCTCATCCGTTCCATATCGGGAAAGACAGAGTATTGGCGGAATCCGGTCTTGTCCCCATTCTCCGGCCAGT
AGCCGCTTCCCAAGGGTCAATGACAGCGCTCCAACAGTTTTCCAGGGATGGGACGTTCCTATCGGAGTAAGTAC
CCCATCGGCTGATTAGACCATAGCTGACGCAGAGCCCTGCAGACCCCGATTAGTATGAACATCTGGAACACGCA
CTACAACAAGGATTTCTTCCCTGGCCCCACCGAATTCTGGCCGGAGCGCTGGATGGGCGAAGGAACTCGGGAG
CTAGAGAAGTATCTAGTTCCATTTGGCAGTGGATCCAGAATGTGTACCGGTCAGAAGTAAGAAACCCACTCCCA
TGCCAGAATACAGCGGAAGGGGGCTCACAAACGACAAACAGTCTATCCATCGCCGAGCAGGTTCTCACCATCG
CCACGCTCTTCCGCAACTACGAGCTTGAGCTGTACCAGACCACCAAGAAAAACGTTGTGATGGCGTCATACTGC
ATGATCTCGCTGCCTGGATCTGAGTCGCCTGGTATTCAAGTGAAAGTCCGCAAGACGGTGCAATAG

A. terreus NIH2624 _atD_ (GenBank accession No. KY950682)

ATGGGTGAACTGTTCAAGTGGCCGAGCTTCTCGCCGTTCGTACCGTTTCACGTGTCTGCTGGGCAGACGGTGAT
CAGAAAGTGGGCATAGAAAGACAACCGTGCAATTCCTTAGTTAACAGATCAACTAATGAACAATGCGCTAGATT
CGGCGGTCTGCTCACCTGCGAATTCCTCCCTCCCCCTCCGGGGCGGAGTTTCATGATGCGACAAACCTACCGCC
ACAGCGTTGAGGGTCCGATCCCGGAGAATCTACGCAAGCTGATCGAGAGCGACCACCGTCCGGATGGTCCGCC
GATGCACTTCCACCAGTGGCAGACAGAATACTTCAAGGTCGAAGAGGGTATCTGTGTTGTGGAAGTGAACGGC
AAGCAGACGATGCTGACCCCCGACGACGAAGAGATCTCATGCAAAGCCGGCAACATCCACCGGTTCTTCATCC
ATCCGGATTCTCGCGAGAGAATGACGGTGATTCTGAGCGCCTCGGATTCCGGCGTGGACTACCAGCTGGACAGA
GTCTTCTTCGAGAACTGGTATGGATATTGGCATGACGCGTTGCTGTACCAGGGCGGCTTGGATTTTATTCAGACC
CTTTGCGTAAGCCCAGATTTTCTCTACTTTCAGCTCTCGCTTGCTAACCAATCTATTTAGATCCACGATGCGGGTG
ACCACTACACGCCCGGGCCCGCCTGGCTGCCATTCCGCCGCTTCATCGGGTACTGGATGTGCGTGGTGATCGGC
CGCTGGATTGGCGGCTTGCTTGGCTACAAGCCGTTTTTCCGGGAGTACACTACCGACTGGGACTTTGCCGTAAC
CAAGATGAAGGCTAATCCCTGGACGCGTCGGCTGGTGAACGACTCATACGCGAACAAGAAGTCCTGGGACGAG
CAGGTGGAGCTGTCCTCGCGCCCCAAGGCCCAGAACGCGGACTACGAGCTGCTGGTGACTGACATAACCGAGG
AGAACCGACGGAAGAAGGCCCAATGGAGCTACGAATGGACATGCGAAATTGGCGAATGGCACCGCTACCGGTG
TGGCAGTCGAGGTGAAGGAGAATGGGGAGGAGCTCCGAAAGCGCTCCTAAGGGGTGAAGGGCTGTTATTATCT
TATGTGCTGCTTGTCTGCTTGTTCCTTTTGTCTGCTTACTGTGATATGCGATGA

A. terreus NIH2624 _atC_ (GenBank accession No. KY950684)

ATGCGCGTGTTTCCCACATACATTGCTGTCTCGGGCCTATTTGGTGGCGCCTTTGCAGCTTTTGGCGCCA
CCAACATCAAAGGCCAGACGAAGCTGTTTGGTACCTCTTTCGGGATTCTTGCAAAGAATGCCTCGTATGA
CTATGTCATTGTTGGAGGAGGCACTGCAGGCTTGACCGTTGCGGCCCGACTGGCCGCACAGCCGAATGTC
TCTGTTGCAGTGATTGAGGCTGGAAGCTTCTATGAAATCGACAATGGAAACATCAGCCAGGTACCTGGAT
ATGGAGCTAACTACTTGTCATTCAATGACCTGACGCCGAGCCCTGTGTTGGTGGATTGGGGATTGATCAC
AGAGCCGCAGGACGTAAGGACACCTCTGATTCATCGTGCGTATTCCTTAACTGACAGATTCCAAGGGCCT
CAACAACCGTCAAATTCATTACTCCGCTGGCAAGACCCTTGGGGGGAGGTTGGTCAACACACCTGGATGT
TTCGTTGGGAGGAAACTAACCGATTGACTTCCAAGCTCTGCTCTGAACGACATGATCTTCCACAGGTAGG
ATCTTCCTATTCATCAATCCATGGTTGGTCACTTTGCTTACGGAGATGGTTAGGGCTACAAAGGGTTCTT
ATCAGCGGTGGGCGGAGCTTGTCGACGACGACACCTACACGTGGGACAAACTACTTCCTTATCTGAAGAA
GAGTGTGGACTTTACCAAGCCTAAGGATGCCGCCACTTATCCTTATGATGCCTCCGTCTACAGCCCAGAG
GGCGGTCCTCTCCAAGTCTCGTTCCCCAATTACCGAGCCCCCTGTGATGACTTCATGGAGACGGCTTTCA
CCAAGTCAGGGCTCAAACCTATCAAAGGCCTTAACAGCGGGCATCTAGATGGCTTCGCACCGACTACATT
TGTGATCAACCCTGCGGACCAAACAAGAAGCTCCTCCGAGGCAGCGTTCCTCCAGGAAGCTCTGGATACC
ACTGCCATGACCCTTTATCTGCGGACTTTAGCGAAGAAGATCCTTTTTGATACCAACAAGACTGCAAATG
GGGTCCTTGTGGAGACTAACGGAGCAGAGTACACCATCTCTGCGAAGAAGGAAGTCATTTTGTCGGCTGG
AGTGGTATGTGAACAAACCGAGGCCCCGACAGACTTCTCAGCTAAAGGAGTATCAAACAGTTCCATTCCC
CTCAACTTCTTCTCCTCTCAGGAATTGGTCAAGCAGATAGCCTCGAGAAGTTTGGAATTCCTGTAATCTC
TGATCTGGCGGGCGTTGGGCAGAACTTGTGGGTAAGTTGTTTGGAGACTCATTCTCAGCGCTAGACTAAA
ATCACCACTAGGATCACCTCTTCATCTTCACCAGTCATGAGATGAATATCACCACCAATTCAGGGGTTCT
GGTCGACCCCGAACTGCTCGCGGAAGCCGTCGAATCGTATCTCAACCAGCAGACCGGTCCACTTACGGGC
ATTGGCGGTGGCGTTGTTGGTGAGTTACCTGCTCCACACGCCATTGTTTAAAGGGGTGTCGGCCTTGCTA
ACTGGATGATGGTAATGTATAGGATGGGAAAAACTCCCCAACCGGGTCAGCTTTTCCAACTCGACAAATG
AAACCCTGGCCAGTTTCCCAGATGATTTCCCCGAAGTGGAATATGTGGCCCTGGCTCCTGGCTCCAACCC
CGCATCCGATCCGCTTGCAAATCACTTTGCCTCCGTCACCGCTGCCGTCCAGAGCACCAGCTCTCGAGGG
TATGTGAAGCTGCGCAGCGCCGATCCCCATGACGCACCCATCATCAACATCAATGCTTTAAGCCACCCTG
CGGATGCCGATCTGGCTGTCGGGGCTATCAAGCGACTGAGACAGATTGCAGAGGCAACTGGAGTCCGCGT
CAAGGAGGTTCTGCCTGGTCCAGAAGTCGTAAGCGACGCTGAGATCTTGGAGTGGGTGAGGAACAACGCC
GTGAATGGGTATCACGCCAGCTCAACATGTATGGCTTTCCACCGAACCACTACACGCAGCAGGCTGTGGG
CTGATCAAACATTTCTAGGTGCAATGGGCAATTCTTCGAATCCGGACGCCGTCGTCGATACTCGCGCCAA
GGTATACGGTGTTTCGAACCTGAGAGTCGTGGATGCGAGTGCTTTGCCGTATCTCCCTCCGGGCCATCCG
ATGAGCTCCATCTGTAAGATGCCCTAAGGCTGTTCGATGAGTGTGGCTAACTCCACTATTTAGATGCATT
TGCCGAATTGATTGCAGAGGATATCTTGAGTAAGTGA

Amino acid sequence for AtA:

MTFSNRVSVAIIGGGIGGLSLAIGLLQNKNLDVAIYETAPKFAEIGAGVALGPNAQHALALISPAAEHAFRI
HATTSLSPEFEHVWFDFRNGNAGEKDGEVLSKVENETGQQTVHRAKFLDELVKLIPREIAHFGKRLIHIQ
KDPVSGGAQYKLFFEDGTTASADCVIGADGIHSSVRKHLLGESHPAATPVFTGTVVYRGLIPMDVARDAI
GEFADNSYMWCGDGGMVMTYPIDHGETLNVVGTRNDKGRWDGPPYTRPVDEETVRNDFMGWGEIPS
KVIQLLKQPTMWAILDHYPAPYYYSGNVAIMGDAAHATTPFQGAGAGQAIEDALVLSTLFQRVTHSGLV
RPALAAYNNVRLRRTQKVVATSRDALRLFCFNDRYVDGDAQRWREVWNGRMDWLWGMDLEKQNRD
AVNLFADIVEKQSSARETMPKFGPVPFPVATSTV

Amino acid sequence for AtE:

MATLPILDLWSTNKALVSLTISASLLLVSLLVSLFQVGRRERGLPPGPPTRLVLGNEHEIPKADSHFLMSK
WAKQYGGIFSLKRFRNTTIVLTDWKIMKELVDKKSTNFSHRPPSKVADLITRGNHILMMQYGETWRTMR
KLIHQYLMESQCEKEHWKVQEAEAAQMLHDFLVDPENHMKHPKRYSNSITMSLVFGIRAKSVNDEYMT
RLYSLMEKWSLVLETGATPPVDSWPLLQWIPERFMGYWRRRATEVGDLMTGLYTEVLHVIENRRKAGI
YKDSLMDRVLDKKDKYRFDEHQLAFLGGTLMEGGSDTSSSLILAIVQAMTQYPEVQKKAHAEIDSVIGT
DRSPAWSDFRKLPYINMMIKEAHRWRPVLPLGVVHGLATDDSYNGMHLPKHSTVILNVWGMHMDPDR
FENPDAFIPERYANFPELAPHYAALADGAARDHFGYGAGRRICPGIHLAERNLFIAVAKLLWAFEFKNNPA
GKNDASAETGSSQGFMHCVKDYDAIVTVRGEERRQTILRELEQAQTVFAKYD

Amino acid sequence for AtG:

MISSVPYRERQSIGGIRSCPHSPASSRFPRVNDSAPTVFQGWDVPIGTPISMNIWNTHYNKDFFPGPTEFWP
ERWMGEGTRELEKYLVPFGSGSRMCTGQNLSIAEQVLTIATLFRNYELELYQTTKKNVVMASYCMISLP
GSESPGIQVKVRKTVQ

Amino acid sequence for AtD:

MGELFKWPSFSPFVPFHVSAGQTVIRKFGGLLTCEFLPPPPGRSFMMRQTYRHSVEGPIPENLRKLIESDH
RPDGPPMHFHQWQTEYFKVEEGICVVEVNGKQTMLTPDDEEISCKAGNIHRFFIHPDSRERMTVILSASD
SGVDYQLDRVFFENWYGYWHDALLYQGGLDFIQTLCIHDAGDHYTPGPAWLPFRRFIGYWMCVVIGR
WIGGLLGYKPFFREYTTDWDFAVTKMKANPWTRRLVNDSYANKKSWDEQVELSSRPKAQNADYELLV
TDITEENRRKKANGATNGHAKLANGTATGVAVEVKENGEELRKRS

Amino acid sequence for AtC:

MRVFPTYIAVSGLFGGAFAAFGATNIKGQTKLFGTSFGILAKNASYDYVIVGGGTAGLTVAARLAAQPNV
SVAVIEAGSFYEIDNGNISQVPGYGANYLSFNDLTPSPVLVDWGLITEPQDGLNNRQIHYSAGKTLGGSSA
LNDMIFHRATKGSYQRWAELVDDDTYTWDKLLPYLKKSVDFTKPKDAATYPYDASVYSPEGGPLQVSF
PNYRAPCDDFMETAFTKSGLKPIKGLNSGHLDGFAPTTFVINPADQTRSSSEAAFLQEALDTTAMTLYLRT
LAKKILFDTNKTANGVLVETNGAEYTISAKKEVILSAGVFHSPQLLLLSGIGQADSLEKFGIPVISDLAGV
GQNLWDHLFIFTSHEMNITTNSGVLVDPELLAEAVESYLNQQTGPLTGIGGGVVGWEKLPNRVSFSNSTN
ETLASFPDDFPEVEYVALAPGSNPASDPLANHFASVTAAVQSTSSRGYVKLRSADPHDAPIININALSHPA
DADLAVGAIKRLRQIAEATGVRVKEVLPGPEVVSDAEILEWVRNNAVNGYHASSTCAMGNSSNPDAVV
DTRAKVYGVSNLRVVDASALPYLPPGHPMSSIYAFAELIAEDILSK

Supplementary Figure S1. The identified gene coding sequence and introns of *atA*, *atE*, *atG*, *atD*, *atC* by reversely transcription analysis and the related translated amino acid sequences. The yellow highlighted regions for each gene were introns; the red font showed the identified 5’ flanking gene coding sequence correcting the previous genome shortgun sequencing results (GenBank accession No. CH476602.1). The GenBank accession numbers shown were related with the identified sequences that we have submitted.

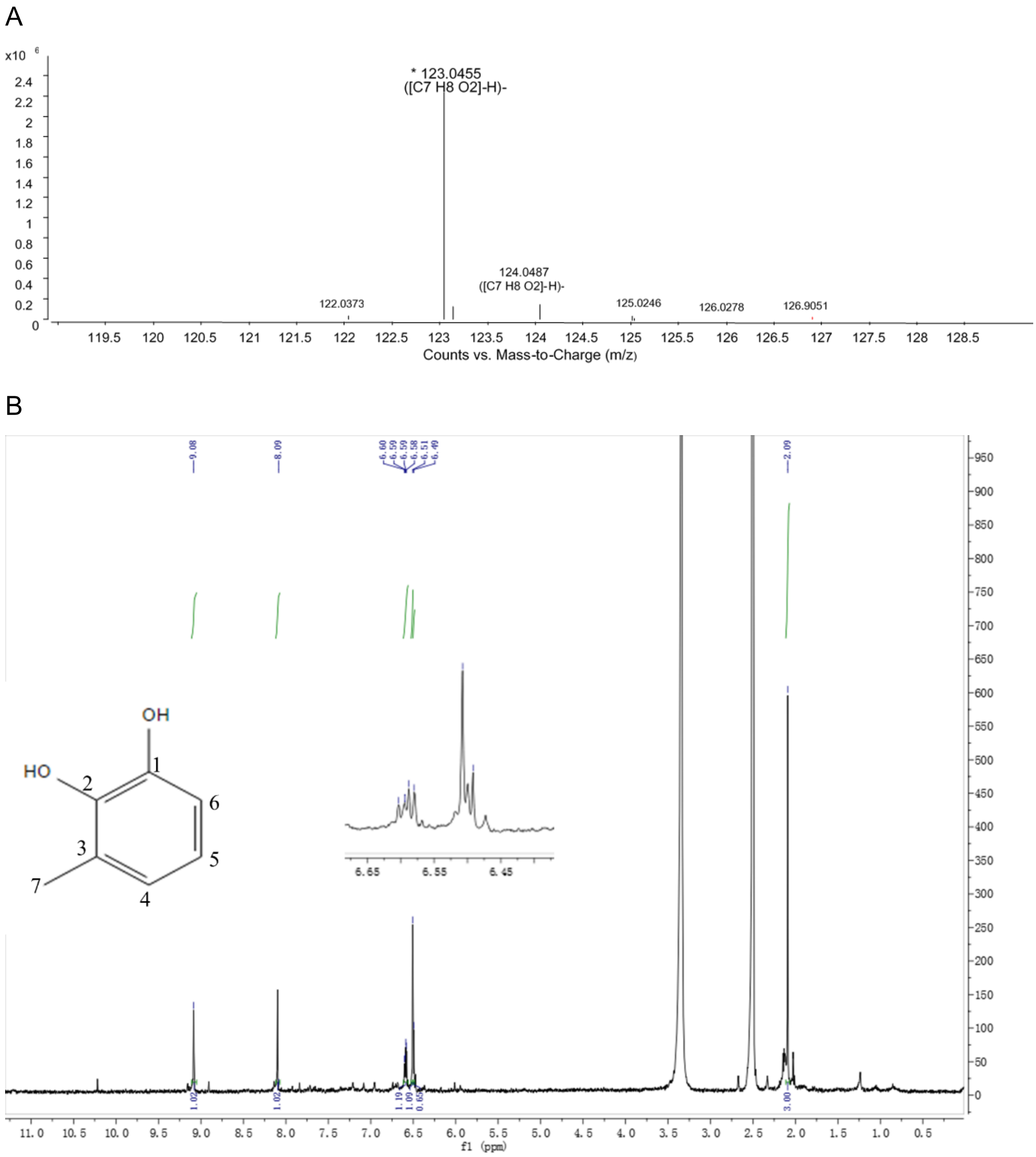

Supplementary Figure S2. LC-MS (A) and ^{1}H NMR (B) for the specific product (compound 5) by GS-NXA. ^{1}H NMR (400 MHz, DMSO-d_6): δ 9.08 (s, 1H, OH-1), 8.10 (s, 1H, OH-2), 6.58~6.60 (dd, 1H, J=5.91, 3.5 Hz, H-5), 6.49~6.51 (m, 2H, H-4, H-6), 2.09 (s, 3H, CH_3-7).

A

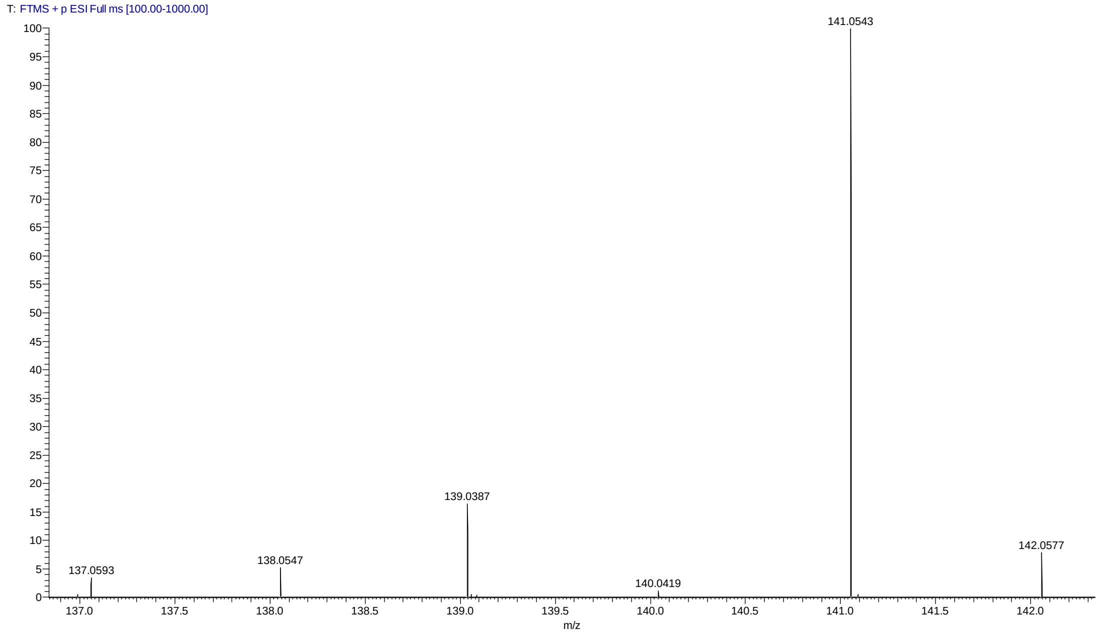
T: FTMS + p ESI Full ms [100.00-1000.00]
141.0543
139.0387
138.0547
137.0593
140.0419
142.0577
m/z

B

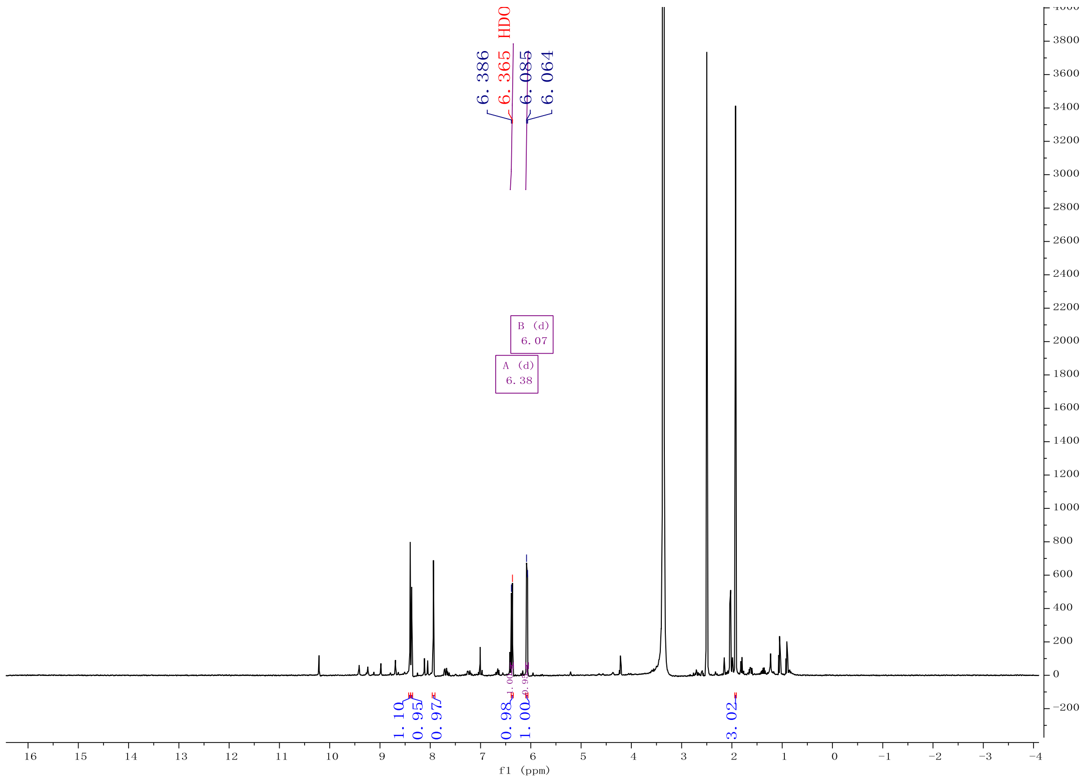
6.386
6.365 HDO
6.085
6.064
B (d)
6.07
A (d)
6.38
1.10
0.95
0.97
0.98
1.00
3.02
f1 (ppm)

C

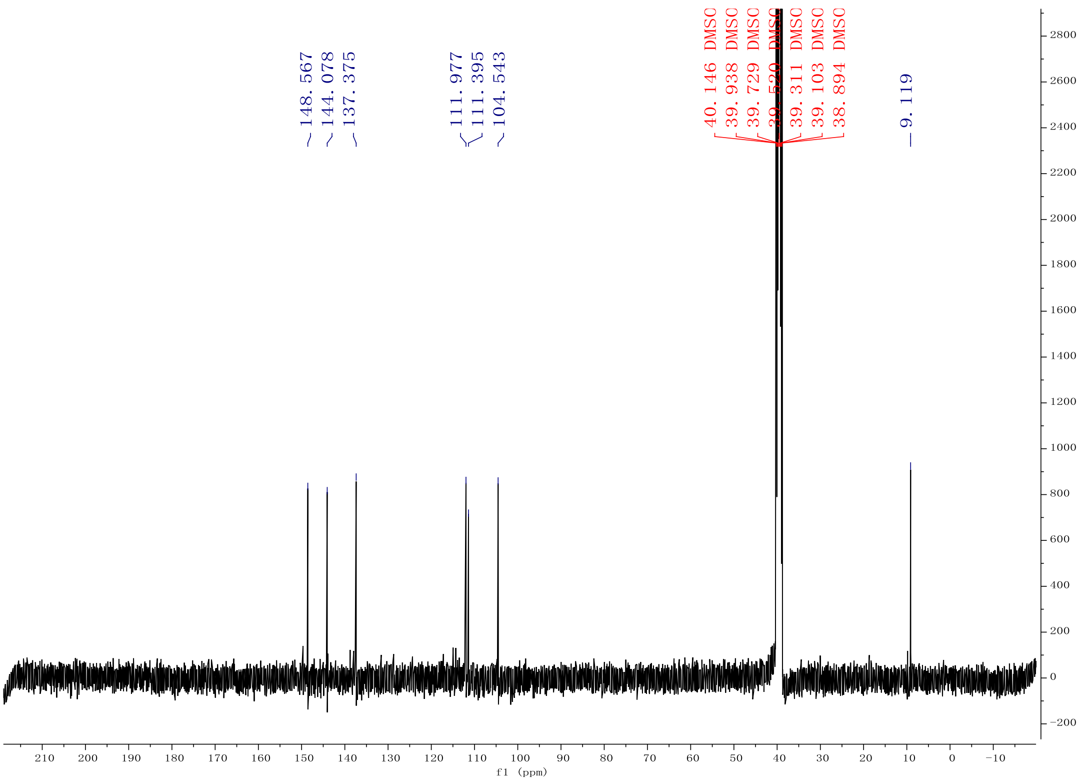
148.567
144.078
137.375
111.977
111.395
104.543
40.146 DMSO
39.938 DMSO
39.729 DMSO
39.311 DMSO
39.103 DMSO
38.894 DMSO
9.119
f1 (ppm)

D

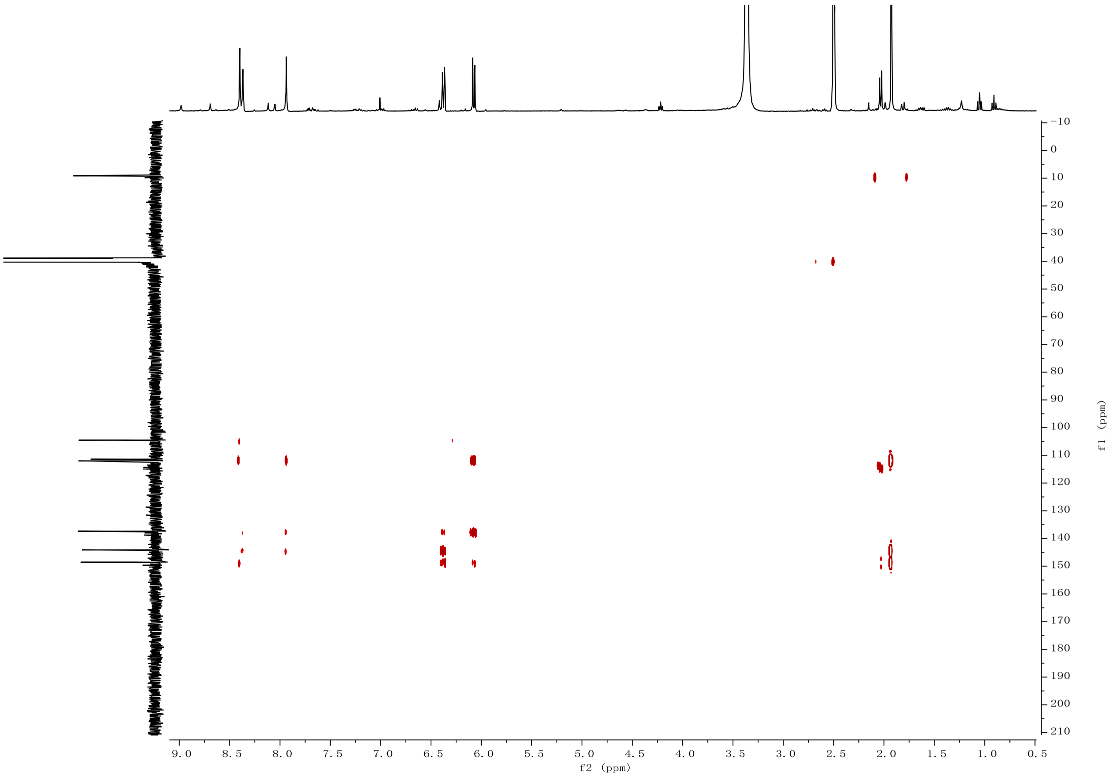
f2 (ppm)
f1 (ppm)

E

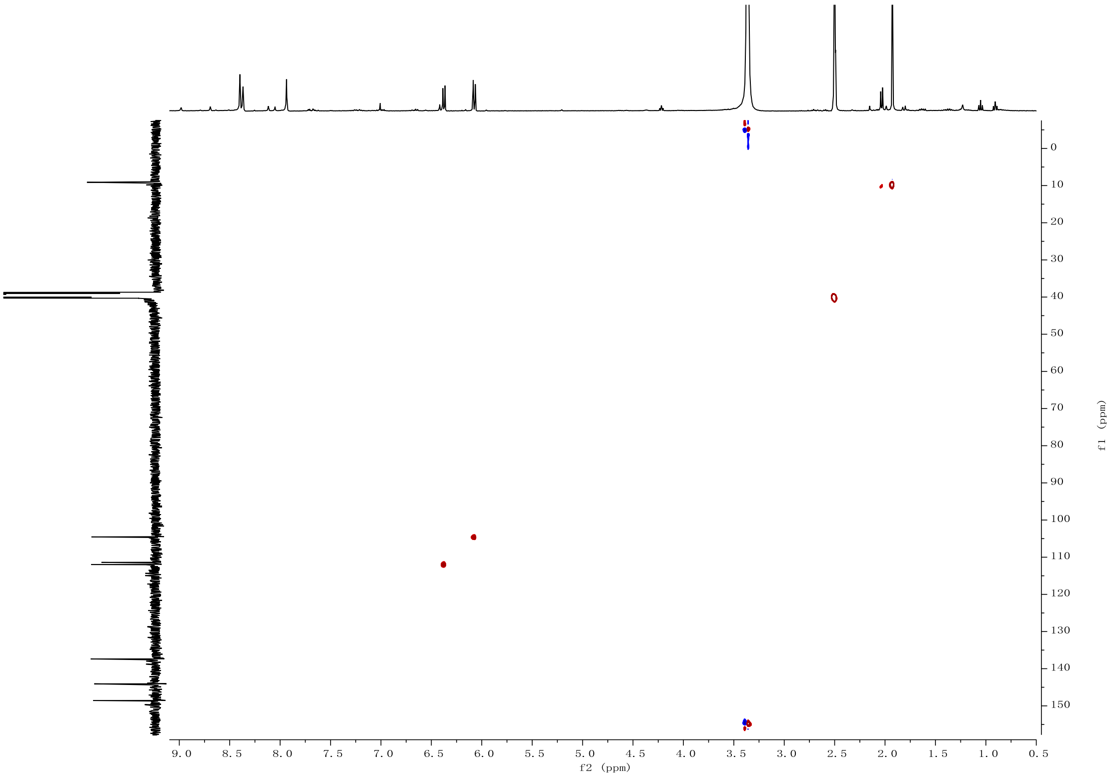

F

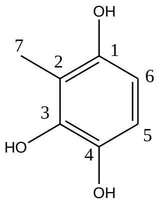

Pos.	δ_H (DMSO-d6) 400 MHz/ppm	δ_C (DMSO-d6) 100 MHz/ppm
1	(OH) 8.49 (s, 1H)	148.6
2	-	111.4
3	(OH) 8.37 (s, 1H)	144.1
4	(OH) 7.94 (s, 1H)	137.4
5	6.38 (d, *J*=8.4 Hz, 1H)	112
6	6.07 (d, *J*=8.5 Hz, 1H)	104.5
7	1.93 (s, 3H)	9.1

Supplementary Figure S3. LC-MS (A), ^{1}H NMR (B), ^{13}C NMR (C), HMBC (D), HSQC (E) and NMR data summary (F) for identification of the specific product (compound 6) by GS-NXAE.

A

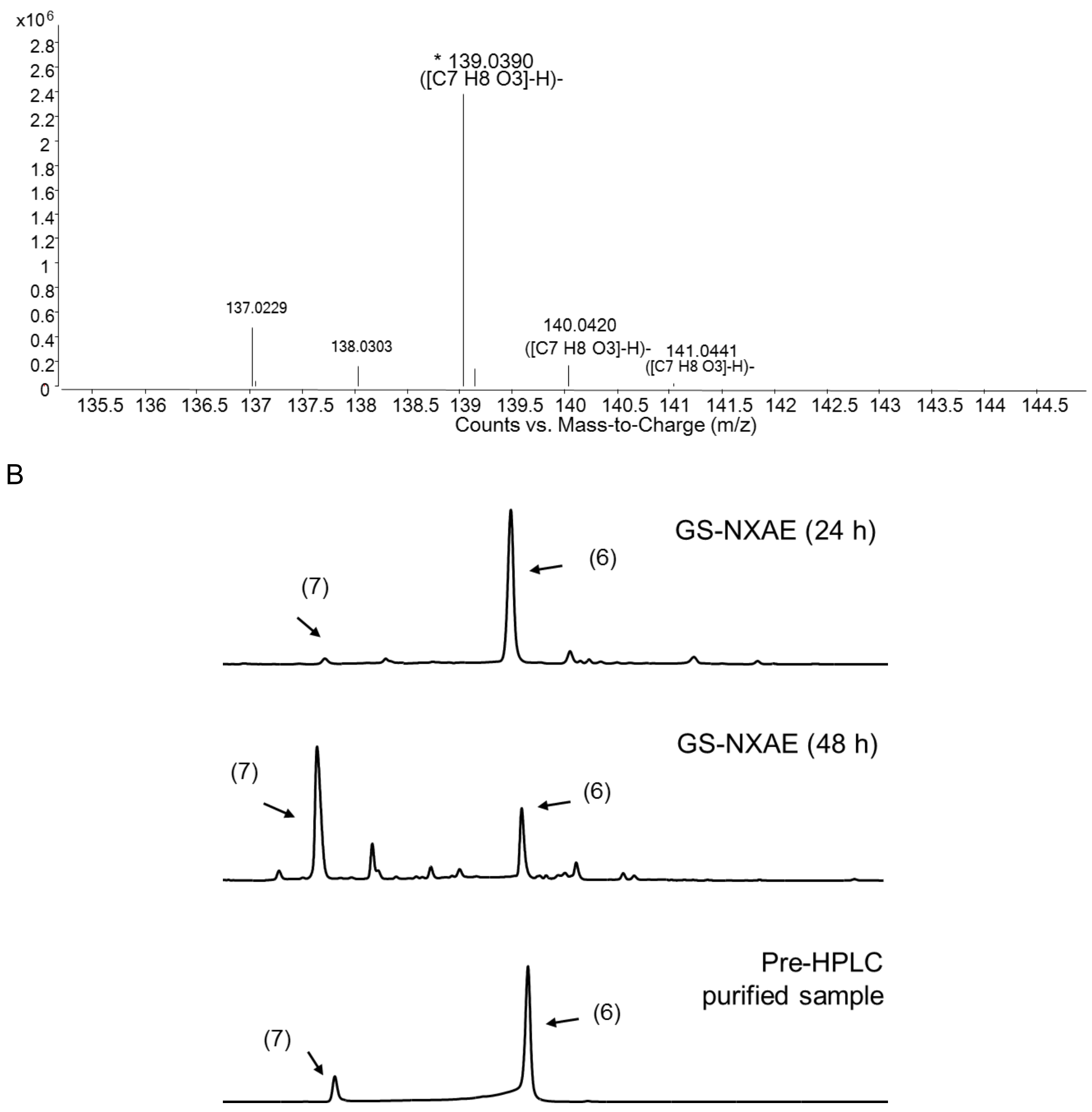

Supplementary Figure S4. LC-MS (A) for predicted compound 7 and HPLC (B) for products from GS-NXAE. Compound **6** partially converted to compound **7** with time going on. Preparative HPLC purified compound **7** immediately transferred to compound **6** before analyzed by analytical HPLC.

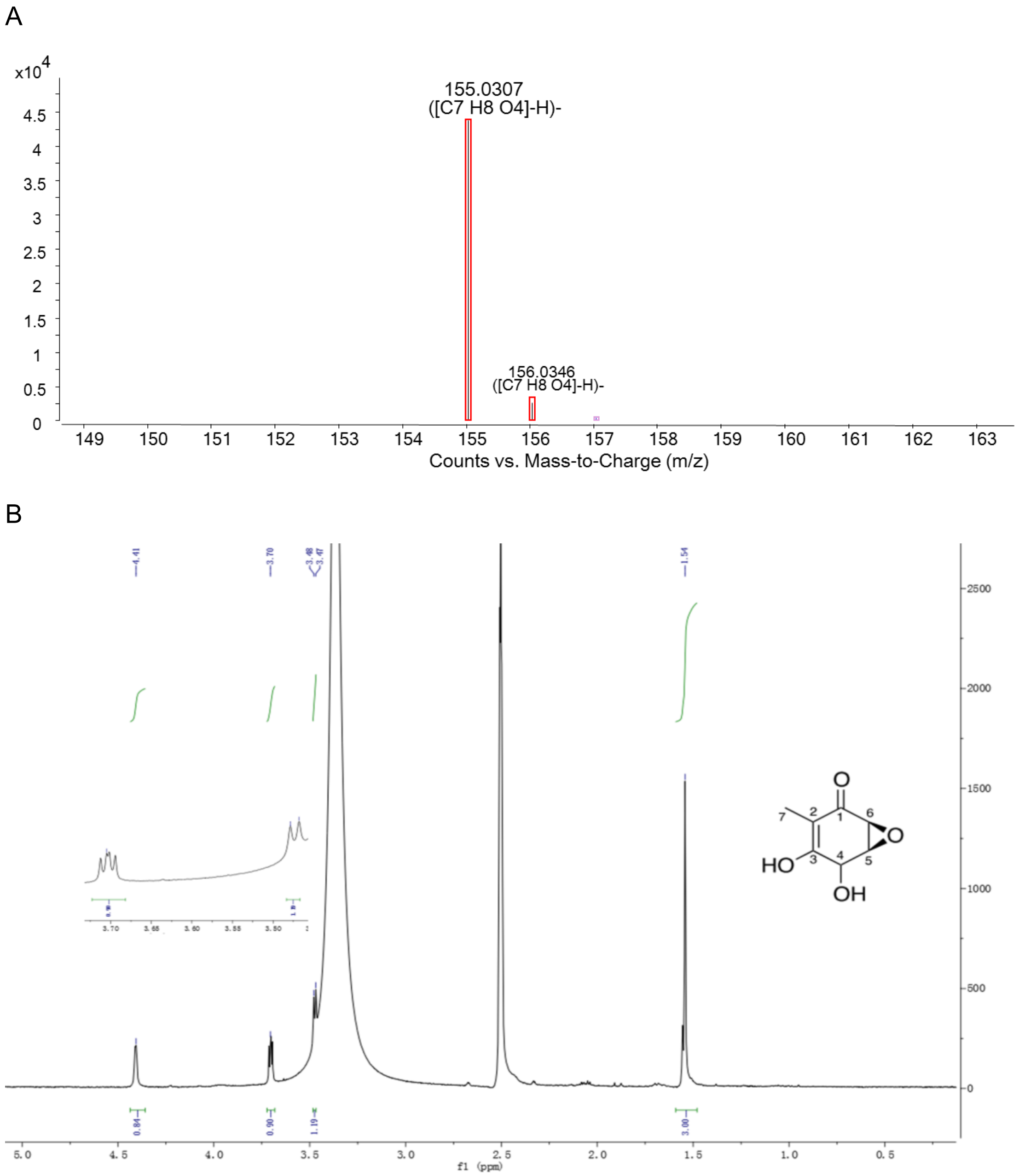

Supplementary Figure S5. LC-MS (A), ^{1}H NMR(B) identification of the specific product (compound 3) by GS-NXAED. ^{1}H NMR (400 MHz, DMSO-d_6): δ 1.54 (s, 3H, CH_3-7), 4.41 (brd s, 1H, H-4), 3.47 (d, 1H, *J*=3.2 Hz, H-6), 3.70 (dd, 1H, *J*=3.2Hz, 1.2 Hz, H-5).

A

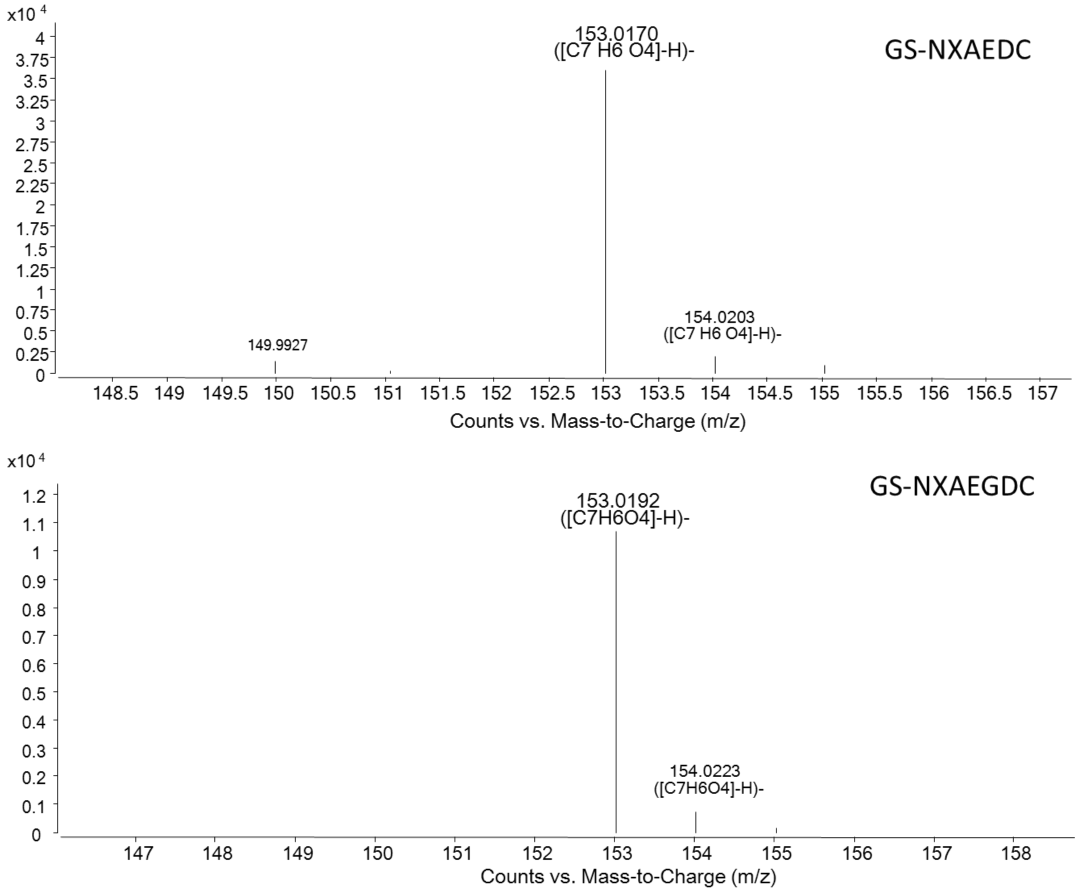

B

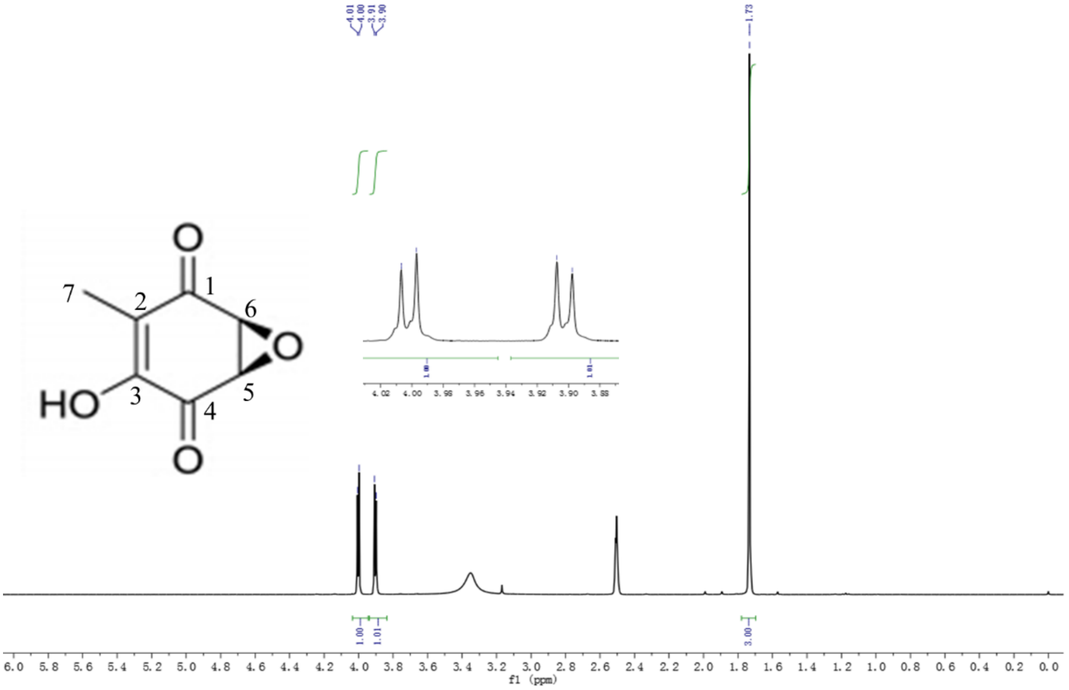

Supplementary Figure S6. LC-MS (A) and ^{1}H NMR (B) identification of the specific product by GS-NXAEDC and GS-NXAEGDC. ^{1}H NMR (400 MHz, DMSO-d_6): δ 4.00 (d, 1H, H-5, J=3.9 Hz), 3.90 (d, 1H, J=3.9 Hz, H-6), 1.73 (s, 3H, CH_3-7)

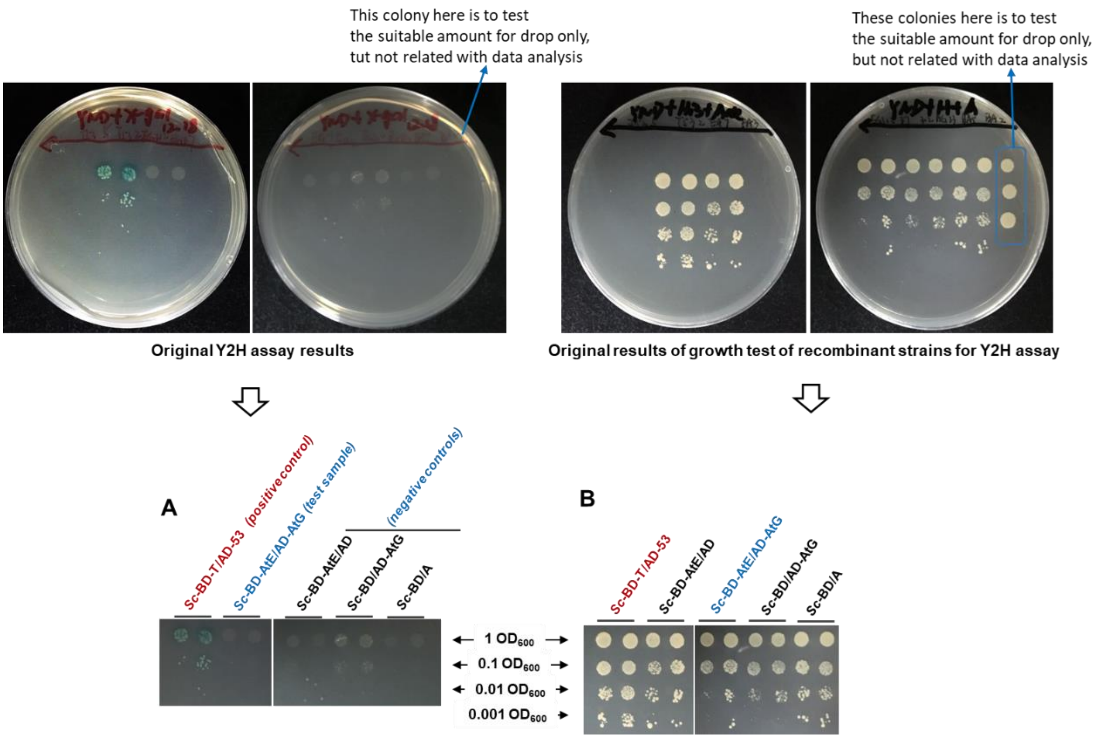

Supplementary Figure S7. Yeast two-hybrid (Y2H) assay for protein–protein interaction of AtG and AtE. (A) Y2H assay for AtG and AtE and positive control and negative controls were checked; **(B)** growth test of recombinant strains on plates free of X-α-gal but with supplemented histidine and adenine for the auxotrophs. A DNA fragment containing *atG* was cloned from the pPIC3.5k-AtG by primers of AD-atG-F/R, and then cloned in frame with the GAL4 activation domain (AD) of the prey vector pGADT7 digested with *EcoR*I and *BamH*I to produce a plasmid pGADT7-*atG*. A DNA fragment containing *atE* was cloned from the pPICZ B-AtE by primers of BK-atE-F/R, and then cloned in frame with the GAL4 DNA binding domain (BD) of the bait vector pGBKT7 vector digested with *Nco*I and *Not*I to produce a plasmid pGBKT7-*atE*. A BD and an AD construct were then co-transformed into Y2H Gold yeast cells following the manufacturer's instructions (Clontech). Standard positive control (pGBKT7-T & pGADT7-53) and various negative controls (pGBKT7 & pGADT7, pGBKT7 & pGADT7-*atG*, pGBKT7-*atE*/pGADT7) were included in the experiment. Strains were verified by genotyping PCRs. Strains with proteins that could bind with each other would grow and show blue colony on YND plate supplemented with X-α-gal. Gradient concentrations of strain broth were dripped in different lines and cultivated for 5 days. Each strain was dripped in two parallels. Strains on plates free of X-α-gal but with supplemented histidine and adenine would grow normally but not show blue colony. Primers, plasmids and strains used in Y2H assay were listed in Table S5 and S6, respectively.

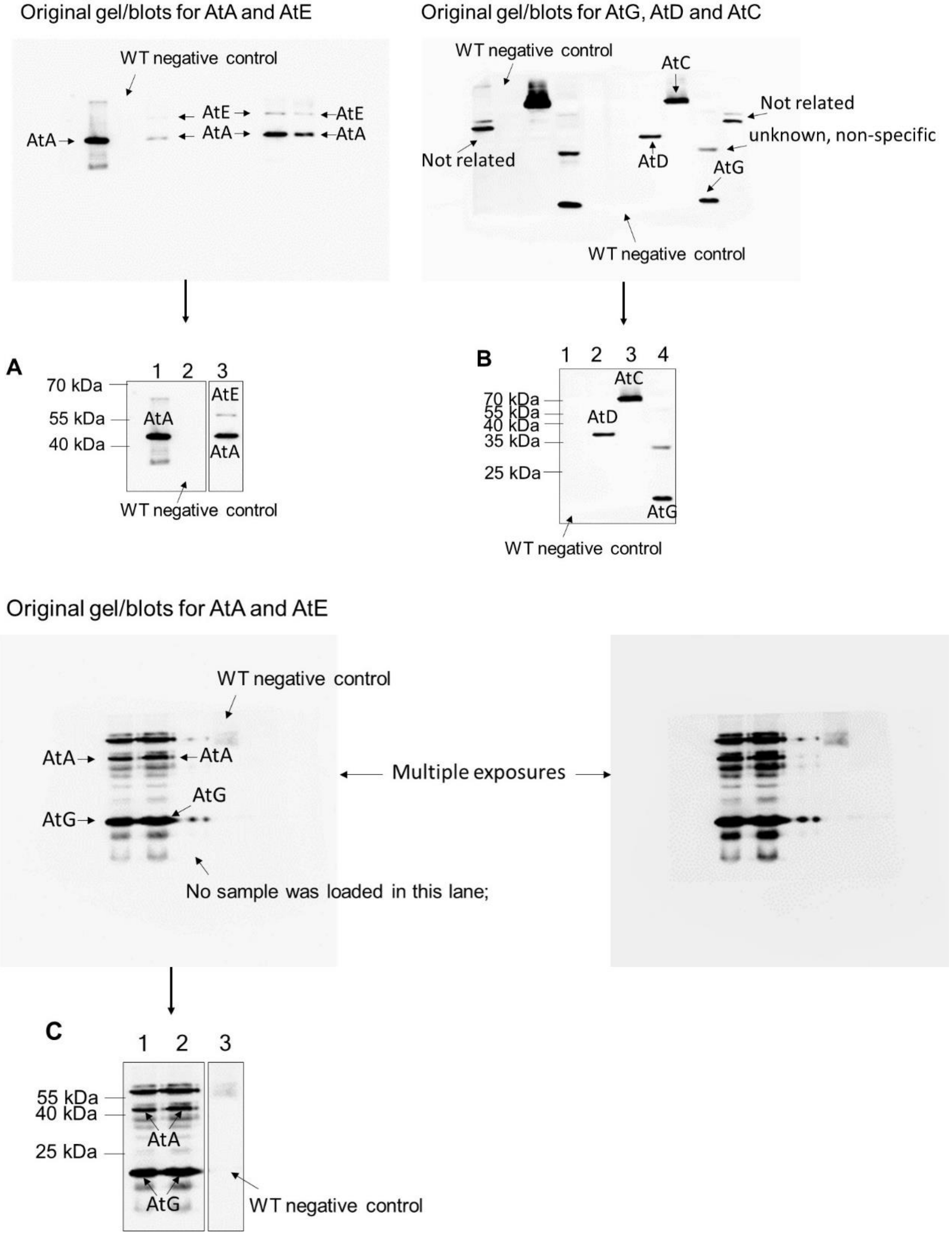

Supplementary Figure S8. Western blot analysis of AtA, AtE, AtD, AtC and AtG expressed in *P. pastoris*. (A) Lane 1: GS-NXA; Lane 2: GS115 as a negative control; Lane 3: GS-NXAE. **(B)** Lane 1: GS115 as a negative control; Lane2: GS115-AtD-HIS$_6$; Lane 3: GS115-AtC-HIS$_6$; Lane 4: GS115-AtG-HIS$_6$; **(C)** Lane 1~2: GS-NXAG; Lane 3: GS115 as a negative control. Original blots were shown and lanes were cropped and reorganized for the three final subfigures. Dividing lines and white space were used to separate cropped lanes.

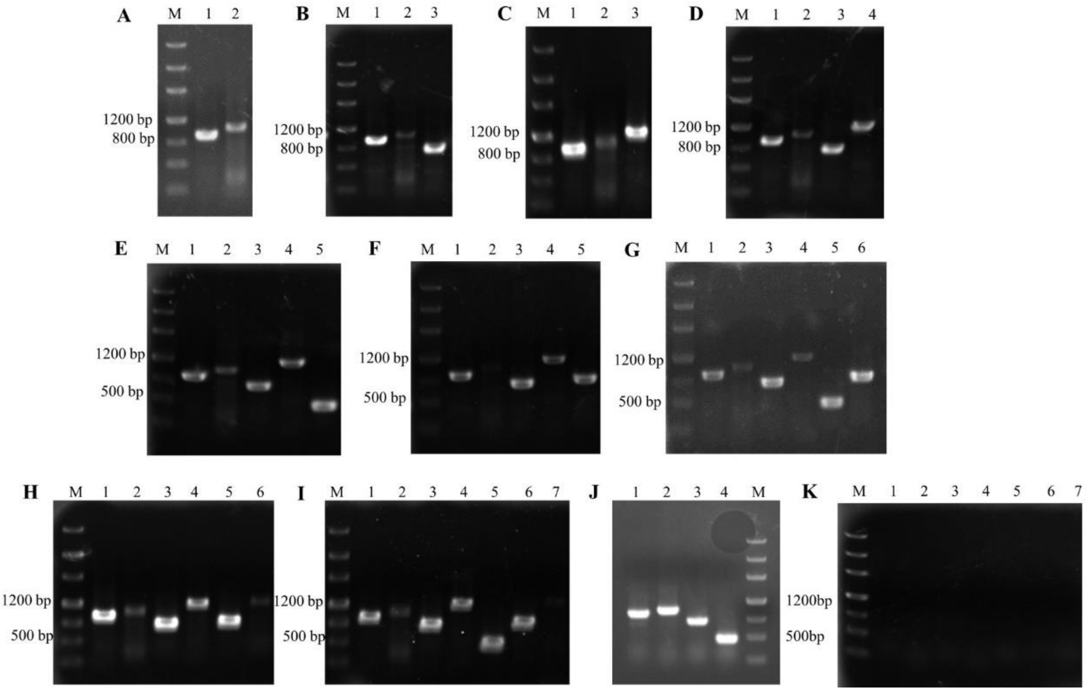

Supplementary Figure S9. **Transcription analysis of terreic acid biosynthetic genes in the recombinant strains and wild type strain GS115 (negative control). (A)** GS-NX; **(B)** GS-NXA; **(C)** GS-NXE; **(D)** GS-NXAE; **(E)** GS-NXAEG; **(F)** GS-NXAED; **(G)** GS-NXAEDG; **(H)** GS-NXAEDC; **(I)** GS-NXAEGDC; **(J)** GS-NXAG; **(K)** GS115. The cDNA were used as templates. Primers for genes were as that, ***Lane 1~2 in (A):*** atX-F/atX-R for *atX*, npgA-F/npgA-R for *npgA*; ***Lane 1~3 in (B):*** atX-F/atX-R for *atX*, npgA-F/npgA-R for *npgA*, atA-F'/atA-R' for *atA*; ***Lane 1~3 in (C):*** atX-F/atX-R for *atX*, npgA-F/npgA-R for *npgA*, atE-F'/atE-R' for *atE*; ***Lane 1~4 in (D):*** atX-F/atX-R for *atX*, npgA-F/npgA-R for *npgA*, atA-F'/atA-R' for *atA*, atE-F'/atE-R' for *atE*; ***Lane 1~5 in (E):*** atX-F/atX-R for *atX*, npgA-F/npgA-R for *npgA*, atA-F'/atA-R' for *atA*, atE-F'/atE-R' for *atE*, atG-F'/atG-R' for *atG*; ***Lane 1~5 in (F):*** atX-F/atX-R for *atX*, npgA-F/npgA-R for *npgA*, atA-F'/atA-R' for *atA*, atE-F'/atE-R' for *atE*, atD-F'/atD-R' for *atD*; ***Lane 1~6 in (G):*** atX-F/atX-R for *atX*, npgA-F/npgA-R for *npgA*, atA-F'/atA-R' for *atA*, atE-F'/atE-R' for *atE*, atG-F'/atG-R' for *atG*, atD-F'/atD-R' for *atD*; ***Lane 1~6 in (H):*** atX-F/atX-R for *atX*, npgA-F/npgA-R for *npgA*, atA-F'/atA-R' for *atA*, atE-F'/atE-R' for *atE*, atD-F'/atD-R' for *atD*, atC-F'/atC-R' for *atC*; ***Lane 1~7 in (I&K):*** atX-F/atX-R for *atX*, npgA-F/npgA-R for *npgA*, atA-F'/atA-R' for *atA*, atE-F'/atE-R' for *atE*, atG-F'/atG-R' for *atG*, atD-F'/atD-R' for *atD*, atC-F'/atC-R' for *atC*; ***Lane 1~4 in (J):*** atX-F/atX-R for *atX*, npgA-F/npgA-R for *npgA*, atA-F'/atA-R' for *atA*, atG-F'/atG-R' for *atG*.

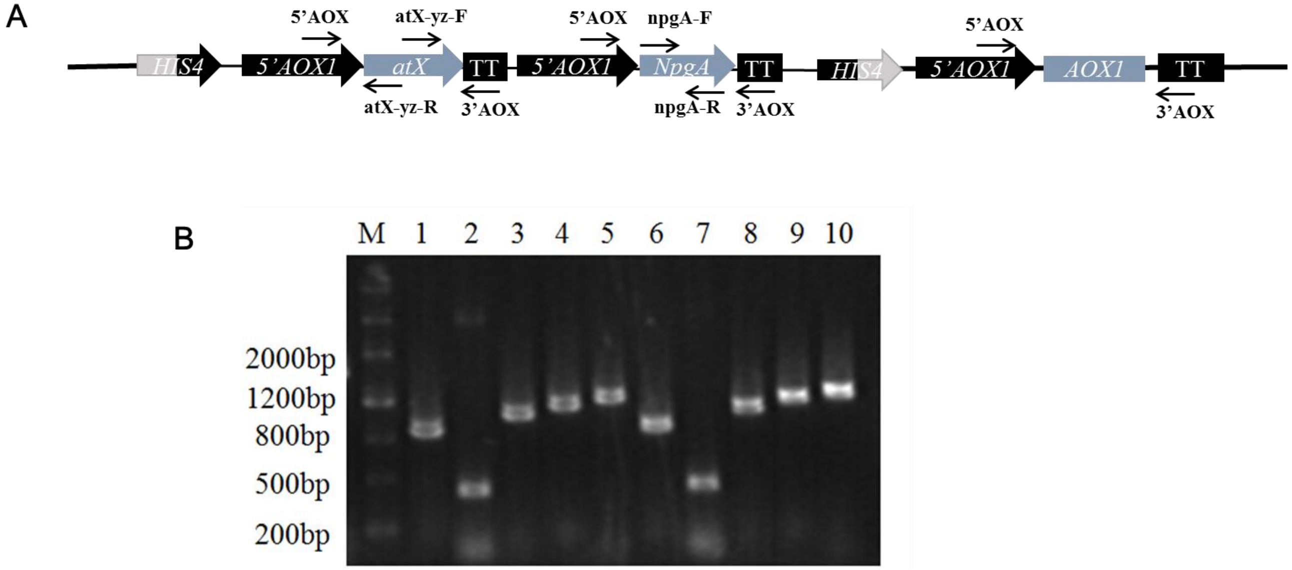

Supplementary Figure S10. Verification of GS-NX by PCR. (A) Scheme for genotype analysis; **(B)** Lane 1~5, 6~10: PCR using genomic DNA of GS-NX transformant and primers 5’AOX/atX-yz-R (870 bp), atX-yz-F/3’AOX (420 bp), npgA-F/npgA-yz-R (1064 bp), 5’AOX/ npgA-R (1151 bp), npgA-F/3’AOX (1159 bp), respectively. Both GS-NX transformants were correct.

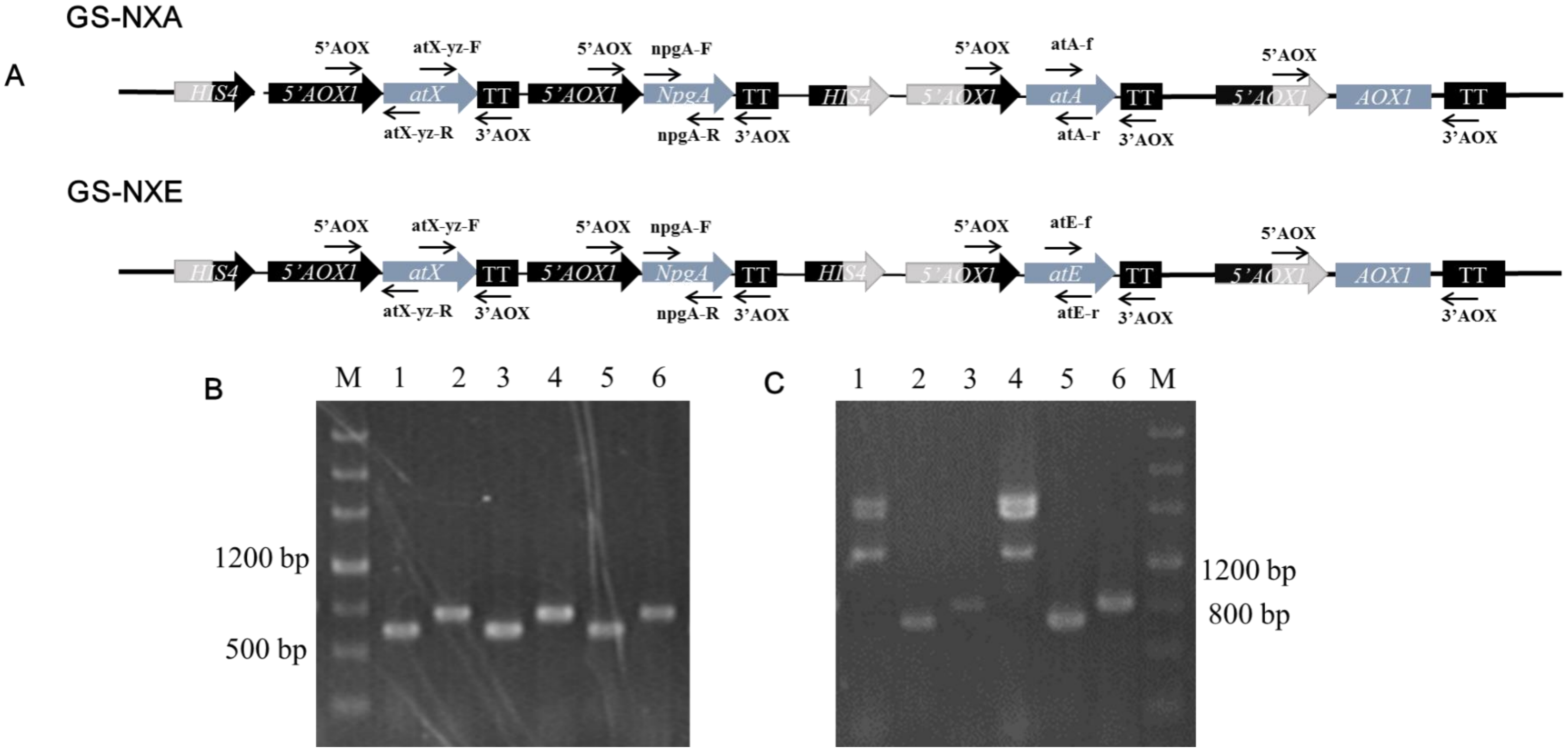

Supplementary Figure S11. Verification of GS-NXA and GS-NXE by PCR. (A) Scheme for genotype analysis; **(B)** GS-NXA. Lane1~2, 3~4, 5~6: PCR using genomic DNA of three GS-NXA transformants and primers 5'AOX/atA-r (655 bp), atA-f/3'AOX (726 bp). **(C)** Lane 1~3, 4~6: PCR using genomic DNA of two GS-NXE transformants and primers 5'AOX/3'AOX (2200 bp and 1900 bp), 5'AOX/atE-r (690 bp), atE-f/3'AOX (801 bp). All strains were correct.

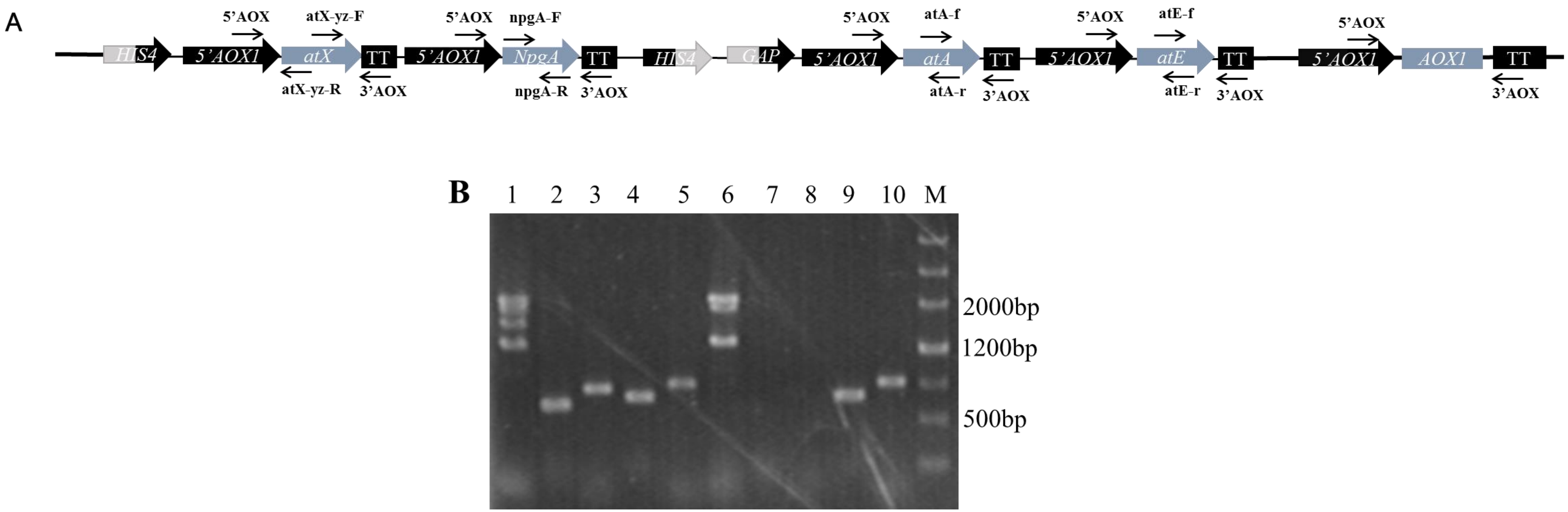

Supplementary Figure S12. Verification of GS-NXAE by PCR. (A) Scheme for genotype analysis; **(B)** Lane 1~5: PCR using genomic DNA of a GS-NXAE transformant and primers 5'AOX/3'AOX (2200 bp, 1900 bp and 1641 bp), 5'AOX/atA-r (655 bp), atA-f/3'AOX (726 bp), 5'AOX/atE-r (690 bp) , atE-f/3'AOX (801 bp), respectively; Lane 6~10: PCR using genomic DNA of another GS-NXAE transformant and primers, indicating this is an incorrect transformant.

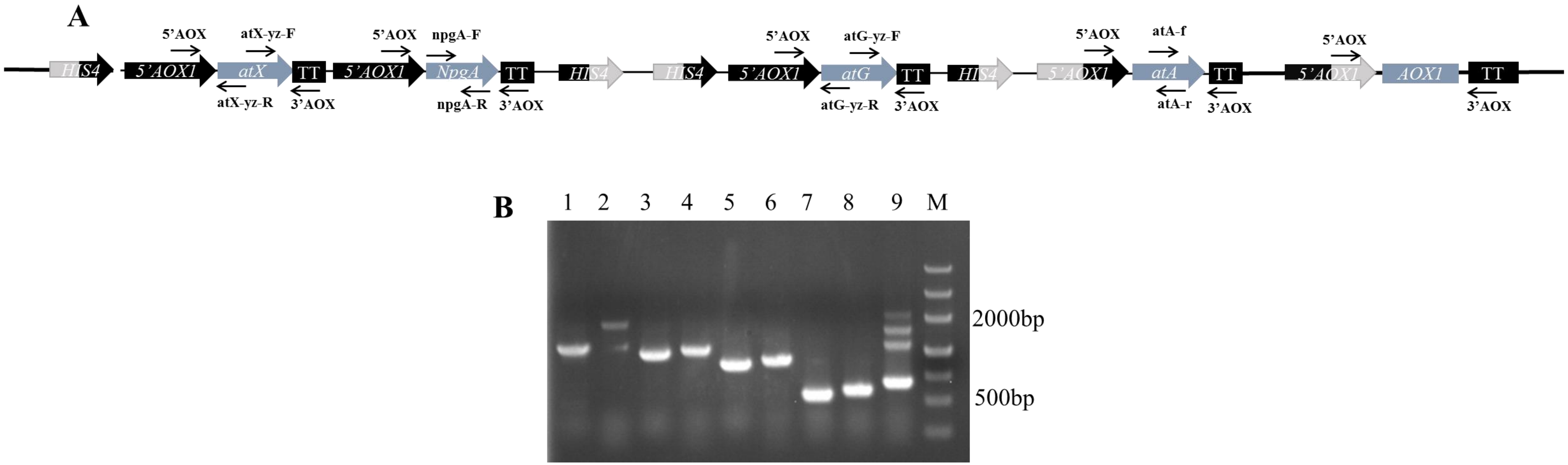

Supplementary Figure S13. Verification of GS-NXAG by PCR. (A) Scheme for genotype analysis; **(B)** Lane 1~5: PCR using genomic DNA of a GS-NXAG transformant and primers 5'AOX/atX-yz-R(1204 bp theoretically), atX-yz-F/3'AOX (1745 bp theoretically), 5'AOX/npgA-R(1053 bp theoretically), npgA-F/3'AOX (1159 bp theoretically), 5'AOX/atA-yz-R(951 bp theoretically), atA-yz-F/3'AOX (1003 bp theoretically), 5'AOX/atG-yz-R(539 bp theoretically), atG-yz-F/3'AOX (645 bp theoretically), 5'AOX/3'AOX (2200 bp,1641 bp,1223 bp and 689 bp theoretically), respectively..

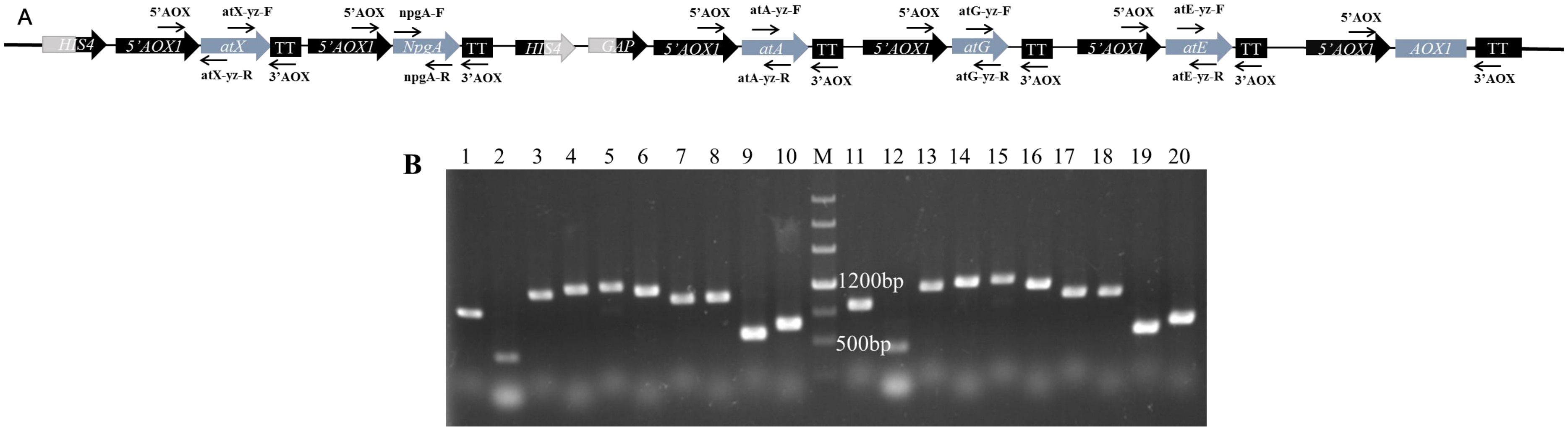

Supplementary Figure S14. **Verification of GS-NXAEG by PCR. (A)** Scheme for genotype analysis; **(B)** Lane1~10, 11~20: PCR using genomic DNA of two GS-NXAEG transformants and primers 5’AOX/ atX-yz-R (870 bp), atX-yz-F/3’AOX (420 bp), 5’AOX/ npgA-R (1151 bp), npgA-F/3’AOX (1159 bp), 5’AOX/atA-yz-R (1239 bp), atA-yz-F/3’AOX (1152 bp), 5’AOX/atE-yz-R (1015 bp), atE-yz-F/3’AOX (1015 bp), 5’AOX/atG-yz-R (554 bp), atG-yz-F/3’AOX (645 bp), respectively. Both strains were correct.

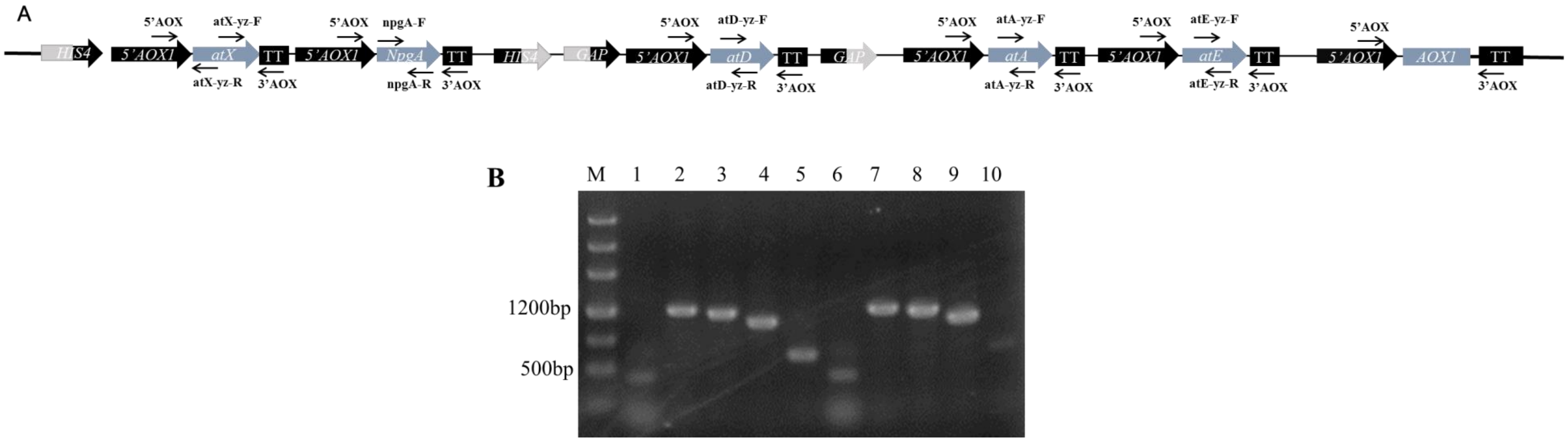

Supplementary Figure S15. Verification of GS-NXAED by PCR. (A) Scheme for genotype analysis; **(B)** Lane1~5, 6~10: PCR using genomic DNA of two GS-NXAED transformants and primers atX-yz-F/3'AOX (420 bp), npgA-F/3'AOX (1159 bp), atA-yz-F/3'AOX (1152 bp), atE-yz-F/3'AOX (1015 bp), atD-yz-F/3'AOX (617 bp), respectively. Both strains were correct.

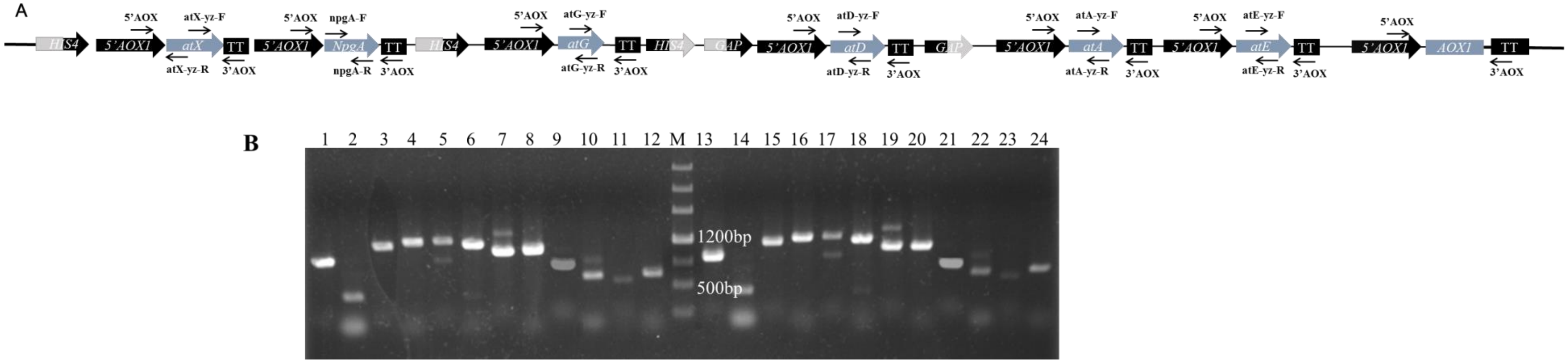

Supplementary Figure S16. Verification of GS-NXAEDG by PCR. (A) Scheme for genotype analysis; **(B)** Lane1~12, 13~24: PCR using genomic DNA of GS-NXAEDG transformants and primers 5'AOX/ atX-yz-R (870 bp), atX-yz-F/3'AOX (420 bp), 5'AOX/ npgA-R (1151 bp), npgA-F/3'AOX (1159 bp), 5'AOX/atA-yz-R (1239 bp), atA-yz-F/3'AOX (1152 bp), 5'AOX/atE-yz-R (1015 bp), atE-yz-F/3'AOX (1015 bp), 5'AOX/atD-yz-R (704 bp), atD-yz-F/3'AOX (617 bp), 5'AOX/atG-yz-R (554 bp), atG-yz-F/3'AOX (645 bp), respectively. Both strains were correct.

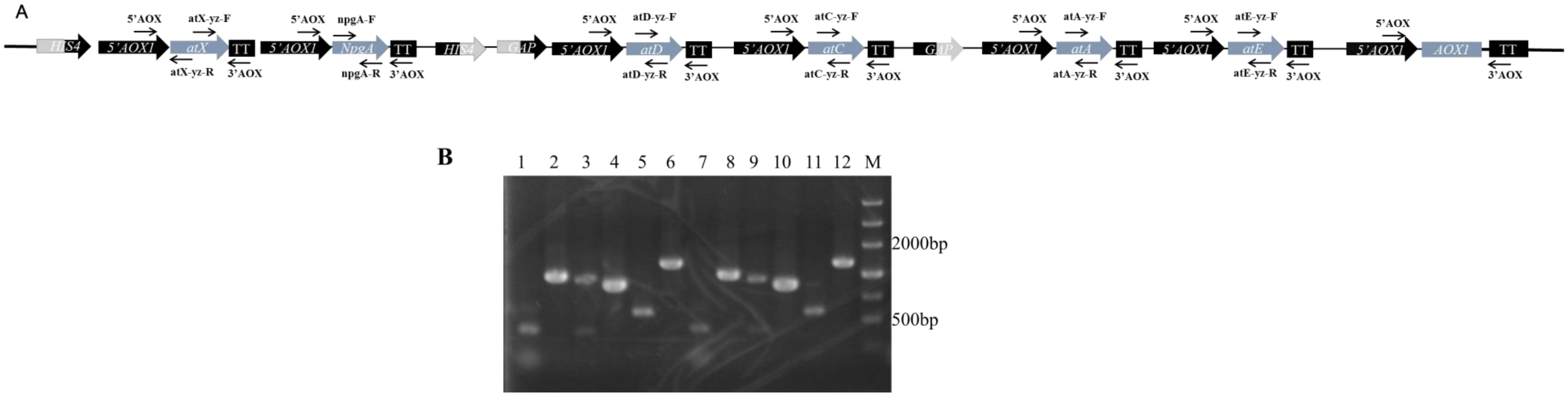

Supplementary Figure S17. Verification of GS-NXAEDC by PCR. (A) Scheme for genotype analysis; **(B)** Lane1~6, 7~12: PCR using genomic DNA of two GS-NXAEDC transformants and primers atX-yz-F/3'AOX (420 bp), npgA-F/3'AOX (1159 bp), atA-yz-F/3'AOX (1152 bp), atE-yz-F/3'AOX (1015 bp), atD-yz-F/3'AOX (617 bp), atC-yz-F/3'AOX (1468 bp), respectively. Both strains were correct.

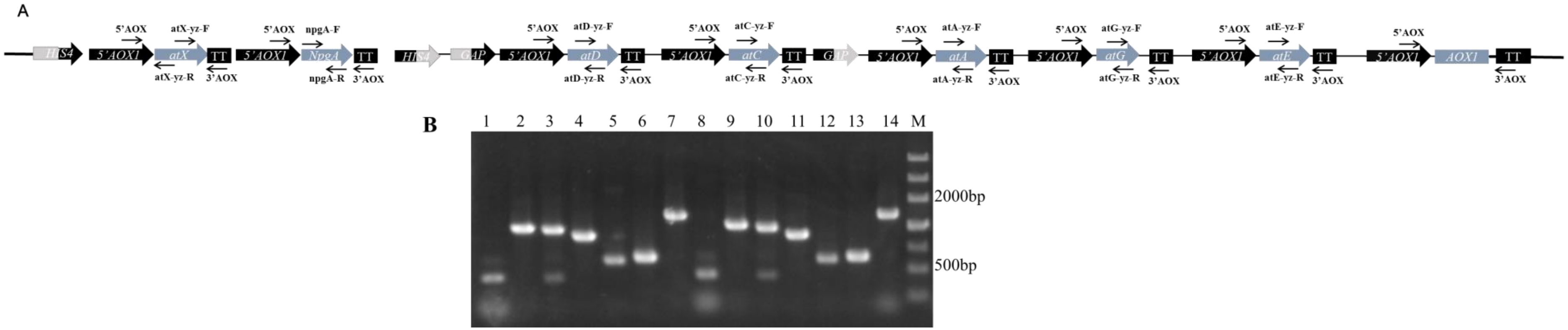

Supplementary Figure S18. Verification of GS-NXAEGDC by PCR. (A) Scheme for genotype analysis; **(B)** Lane1~7, 8~14: PCR using GS-NXAEGDC two transformants DNA and primers atX-yz-F/3'AOX (420 bp), npgA-F/3'AOX (1159 bp), atA-yz-F/3'AOX (1152 bp), atE-yz-F/3'AOX (1015 bp), atD-yz-F/3'AOX (617 bp), atG-yz-F/3'AOX (645 bp), atC-yz-F/3'AOX (1468 bp), respectively. Both strains were correct.
